# Gapless Complete Genome and Multi‐Omics Illuminate Immune Adaptation and Host‐Virus Interactions in a Coronavirus Reservoir

**DOI:** 10.1002/advs.78422

**Published:** 2026-10-28

**Authors:** Yuting Wang, Sheng Zhang, Linxia Ouyang, Xin Du, Xin Huang, Zhilin Wang, Huixian Xie, Jiale Tang, Liyi Lou, Wenqing Ling, Weihao Wang, Jiang Zhou, Zhen Liu, Yibo Hu, Fuwen Wei, Guangping Huang

**Affiliations:** ^1^ Jiangxi Provincial Key Laboratory of Conservation Biology College of Forestry Jiangxi Agricultural University Nanchang China; ^2^ State Key Laboratory of Animal Biodiversity Conservation and Integrated Pest Management Institute of Zoology Chinese Academy of Sciences Beijing China; ^3^ University of Chinese Academy of Sciences Beijing China; ^4^ School of Karst Science Guizhou Normal University Guiyang China; ^5^ State Key Laboratory of Genetic Evolution & Animal Models Kunming Institute of Zoology Chinese Academy of Sciences Kunming China

**Keywords:** evolutionary adaptation, host–virus interactions, immune adaptation, multi‐omics functional analyses, telomere‐to‐telomere genome

## Abstract

Bats are major reservoirs of emerging viruses, including severe acute respiratory syndrome‐related coronaviruses, yet they exhibit remarkable tolerance to viral infections with limited clinical manifestations‐a longstanding immunological paradox. Here, we generate a telomere‐to‐telomere (T2T) de novo genome assembly for *Rhinolophus sinicus* (*R. sinicus*), designated T2T‐RSC1.0 (2.14 Gb), comprising 17 fully anchored autosomes and both sex chromosomes. This gapless assembly resolves 129.19 Mb of previously unresolved regions (PURs) and enables annotation of 848 previously unannotated genes, including 131 protein‐coding genes and five pseudogenes on the 33.75 Mb Y chromosome. Using 2.12 × 10^5^ newly identified single‐nucleotide polymorphisms within PURs, population genomic analyses resolve three major genetic groups and provide improved resolution of genome‐wide population structure. Comparative analyses further reveal population‐level associations between major histocompatibility complex polymorphism and viral diversity. Multi‐omics and functional analyses reveal a coordinated evolutionary remodeling of antiviral defense, antigen presentation, cytokine signaling, and inflammatory regulation in horseshoe bats. This study provides an unparalleled genomic resource for *R. sinicus* and establishes an integrated framework for investigating immune evolution, long‐term host–virus coexistence, and mammalian adaptive evolution.

## Graphical abstract

We present a complete T2T genome assembly for a key bat reservoir. Integrating population genomics, comparative genomics, transcriptomics, single‐cell omics, and functional assays, we resolve previously inaccessible genomic regions and identify evolutionary signatures associated with immune adaptation. This study provides a multi‐omics framework for investigating immune adaptation, host–virus interactions, viral ecology, and pathogen persistence in wildlife reservoirs.

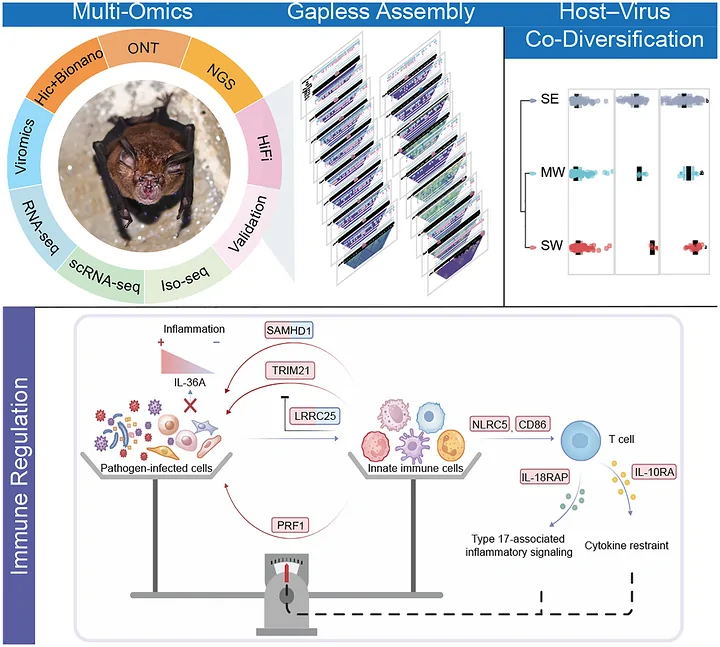

## 1 Introduction

Bats (Chiroptera) represent a remarkable clade within mammals, distinguished by key evolutionary innovations such as powered flight, echolocation, exceptional longevity, and unique immunity [1, 2]. Bats play key ecological roles, such as pollinating tropical flora, dispersing seeds, and regulating insect populations. Thus, they are critical to ecosystem health [3]. From a public health standpoint, bats serve as natural reservoirs for diverse pathogens, including coronaviruses, filoviruses, and paramyxoviruses; however, the bats typically remain asymptomatic [4, 5, 6]. Our understanding of the molecular underpinnings of these traits remains limited.

Considerable efforts have been made to decode bat genomes to unravel bat evolutionary and physiological adaptations. For example, the Bat1K genome consortium aims to generate high‐quality chromosome‐scale genome assemblies for ∼1300 extant bat species [7, 8, 9, 10]. Although high‐quality chromosome‐scale bat genome assemblies have been continuously released in recent years, most of these assemblies still contain unresolved gaps within repetitive, centromeric, segmental duplication, and complex immune gene clusters, which hinders the full characterization of structural variants and immune‐related loci in bats [11, 12].

*R. sinicus* is a widespread and genetically diverse horseshoe bat endemic to East Asia and serves as a natural reservoir of severe acute respiratory syndrome (SARS)‐related coronaviruses [11, 12, 13, 14]. Extensive ecological, virological, and population genetic studies have revealed substantial genetic diversity and lineage differentiation within *R. sinicus*, highlighting its value for investigating bat evolution, immune adaptation, and host–virus interactions. Notably, *R. sinicus* has been proposed as one of the representative bat species for establishing experimental research models [2, 14]. However, existing chromosome‐level assemblies of *R. sinicus* remain incomplete, with unresolved gaps in repetitive sequences, candidate centromeric regions, segmental duplications, and immune‐associated genomic regions. The highly repetitive nature of bat genomes and the complexity of sex chromosomes further underscore the need for telomere‐to‐telomere genome assemblies to resolve previously inaccessible genomic features. The achievement of telomere‐to‐telomere (T2T) genome assemblies in humans [15], apes [16], macaques [17], mice [18], sheep [19], and goats [20] has demonstrated the feasibility of resolving previously inaccessible genomic regions and opened new opportunities for investigating genome evolution in non‐model organisms [21].

In this study, we generated a complete genome assembly of *R. sinicus* and validated the integrity and quality of the assembly. Furthermore, we revealed the genetic differentiation and virome landscapes within its populations. Additionally, we established a single‐cell splenic transcriptomic atlas of *R. sinicus*. Through comparative genomics and in vitro functional assays, we identified candidate immunological features potentially associated with host–virus interactions and immune adaptation in Rhinolophus bats. Overall, these highly comprehensive genetic resources represent a meaningful advance in understanding how natural reservoir hosts coexist with pandemic‐potential viruses, offering far‐reaching implications for future research on the evolution and immunology of this non‐model species.

## 2 Results

### 2.1 Assembly of a Complete Gap‐Free Genome for *R. sinicus*, T2T‐RSC1.0

Assembling the genomes of wild species, such as bats, remains a challenge. To construct a T2T reference genome for *R. sinicus*, we generated a multi‐platform sequencing dataset comprising 247.1 Gb (115× coverage) of ultra‐long Oxford Nanopore Technologies (UL‐ONT) reads, 121.2 Gb (56×) of PacBio high‐fidelity (HiFi) reads, 217.1 Gb (101×) of Hi‐C data, 1.21 Tb of Bionano data, and 126 Gb (58×) of short‐read data from the MGISEQ platform (Figure 1A; Table S1). Three initial assemblies were generated: one using only HiFi data via hifiasm (default parameters), yielding a 2.15 Gb genome with an N50 of 101.99 Mb in 191 contigs; a hybrid assembly combining both HiFi and UL‐ONT data, also using hifiasm, which produced a 2.14 Gb genome with an N50 of 115 Mb in 88 contigs; and an assembly constructed solely with UL‐ONT data using NextDenovo, resulting in a 2.12 Gb genome with an N50 of 82.3 Mb in 62 contigs (Table S2). Although the UL‐ONT‐only assembly resulted in the fewest contigs, we selected the hybrid assembly as the backbone for subsequent analyses, as it achieved a superior contig N50. This hybrid assembly was then scaffolded into 19 pseudochromosomes–corresponding to 17 autosomes plus chromosomes X and Y–using Hi‐C data (Figure 1B; Figure S1A; Table S3). The chromosomal count aligned with previously published karyotypic observations [22].

**FIGURE 1:**
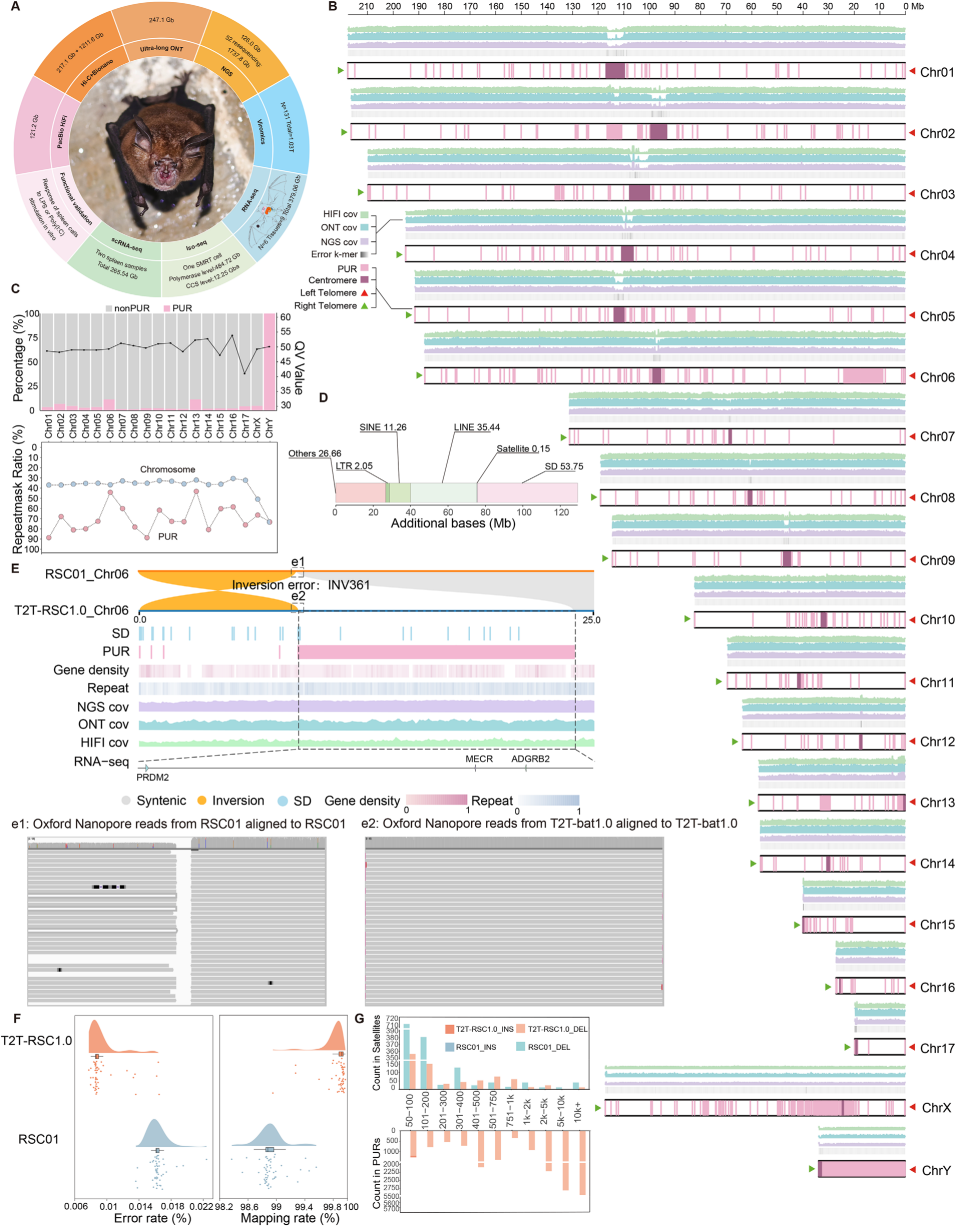
The complete genome assembly of *Rhinolophus sinicus*, T2T‐RSC1.0, and comparison with the previous assembly, RSC01. (A) Overview of multi‐omic and sequencing datasets generated for *R. sinicus* in this study, including PacBio HiFi, Bionano, and Hi‐C data, with additional ultra‐long ONT, NGS, transcriptomic, single‐cell, functional, population resequencing, and virome datasets. (B) Genomic features of T2T‐RSC1.0. The coverages of PacBio HiFi (HIFI cov), ultra‐long ONT (ONT cov), and NGS (NGS cov) short reads are shown in 100‐kb windows in mint green, aqua blue, and lavender, respectively. Error k‐mers (21‐mer errors resulting from the worst assembly) are highlighted in gray. Previously unresolved regions (PURs) in T2T‐RSC1.0 versus RSC01 are shown in pink. Centromeres are highlighted in plum. Right and left telomeres are marked with red and green triangles, respectively. (C) Percentage of PURs (upper) and repetitive elements (lower) in each chromosome. The QV value curve according to NGS reads is shown in the upper region. (D) Length of each sequence type in the PURs of T2T‐RSC1.0. (E) An inversion error (INV361), highlighted in orange, was found on chromosome 6 (Chr06) in RSC01 and corrected in T2T‐RSC1.0. The misjoined junction of this false‐positive inversion cannot be covered by ONT reads from RSC01. One gap containing three genes (*PRDM2*, *MECR*, and *ADGRB2*) with transcriptional expression following INV361 in RSC01 was filled in T2T‐RSC1.0. The coverage of ONT, PacBio HiFi, and NGS reads confirmed the gap‐filling reliability. Gene density, segmental duplication (SD), and repeat density values were calculated in 100‐kb windows. (F) Error rate and mapping rate between mismatches and bases mapped were calculated according to the alignments of resequencing data from 52 individuals to T2T‐RSC1.0 and RSC01, respectively. (G) Numbers of INS/DELs in PUR and satellite regions derived from resequencing alignments of 52 individuals, with PUR regions evaluated using the telomere‐to‐telomere assembly only.

The initial assembly produced 10 gap‐free chromosomes (chr1–5, 8, 9, and 11–13), whereas the remaining nine chromosomes contained gaps ranging from 12.9 kb to 76.35 Mb (Table S4), leaving 129.19 Mb of unresolved sequence (Table S5). These gaps were filled using a combination of UL‐ONT read‐based extension and local reassembly strategies. The accuracy and continuity of the assembly were validated using Bionano optical maps (Figure S1B). Ultimately, all gaps were closed, as supported by alignments of Bionano maps, UL‐ONT, HiFi, and Hi‐C reads (Figure S2; Table S6). The assembly demonstrated uniform coverage across sequencing platforms, with UL‐ONT, HiFi, and short‐read data achieving mapping rates of 99.81%, 99.97%, and 99.48%, respectively, and a base accuracy exceeding 99.999% (Figure 1B; Table S7). Notably, we performed complete assembly of the X and Y chromosomes using hifiasm with haplotype‐resolved contigs derived from UL‐ONT and HiFi reads, supplemented by Hi‐C scaffolding, yielding full‐length sequences of 117.03 and 33.75 Mb, respectively (Figure 1B; Table S6). After five iterative rounds of polishing with UL‐ONT, HiFi, and short‐read data, we produced a complete T2T genome assembly, designated T2T‐RSC1.0, with a total size of 2.14 Gb, slightly larger than the k‐mer‐based genome size estimate (Figure S3A). The assembly comprises 17 autosomes and two sex chromosomes (chrX and chrY) and includes 38 telomeres and 19 centromeres (Figure S3B; Tables S8–S10).

### 2.2 Enhanced Continuity and Accuracy of T2T‐RSC1.0 Over Previous Reference RSC01

The T2T‐RSC1.0 assembly demonstrated a substantial improvement over the previous *R. sinicus* reference genome, RSC01 (RefSeq: GCF_036562045.2), with contig N50 increasing by 3.37‐fold to 187.51 Mb (compared with 42.9 Mb in RSC01) (Table S11). We successfully resolved 129.19 Mb of previously unresolved regions (PURs) across all the chromosomes. Notably, these newly assembled regions are highly enriched in repetitive elements (43.2%‐88.7%), compared to the genomic average of ∼30%–37%, presenting a primary challenge for previous assembly attempts (Figure 1C; Table S12). These PURs were found to comprise centromeric satellites (146.97 kb, 0.11%), segmental duplications (SDs; 53.75 Mb, 41.6%), and repetitive elements, including long interspersed nuclear elements (LINEs; 35.44 Mb, 27.43%), short interspersed nuclear elements (SINEs; 11.26 Mb, 8.72%), and long terminal repeats (LTRs; 2.05 Mb, 1.59%) (Figure 1D). Notably, chromosome Y (33.75 Mb) exhibited the greatest accumulation of PURs, followed by chr6 (21.6 Mb), chr2 (15.1 Mb), and chr13 (6.63 Mb) (Figure 1C; Table S5). Furthermore, T2T‐RSC1.0 was found to provide a more accurate representation of SDs, constituting 17.29% of the genome, representing an increase from the 3.62% detected in RSC01 (Tables S5, S11).

Furthermore, we conducted multiple evaluations to assess the completeness, contiguity, and accuracy of the T2T‐RSC1.0 genome assembly. The average genome‐wide consensus quality value (QV) was 49.14 according to short‐read data (ranging from 41 to 53.87) and 59.72 according to HiFi reads (ranging from 41.91 to 82.66) (Figure 1C; Table S6). The collinearity analysis between T2T‐RSC1.0 and RSC01 demonstrated high consistency and improved assembly continuity in T2T‐RSC1.0, while also correcting 170 structural misassemblies (inversions) present in RSC01 (Figure S4A; Table S13). For example, a large inversion on chromosome 6 (INV361) initially assembled in RSC01 was inconsistent with the raw ONT read alignments, which showed no coverage across the misjoined junctions (Figure 1E). In contrast, alignment against T2T‐RSC1.0 revealed continuous read coverage across these regions, confirming the corrected assembly (Figure 1E).

The INV361 misassembly on chr6 in RSC01 resulted in a downstream PUR encompassing three biologically important genes (Figure 1E; Table S14): PRDM2 (a tumor suppressor involved in histone methylation and DNA repair) [23], MECR (essential for mitochondrial fatty acid synthesis and potentially linked to metabolic adaptation in flight) [24], and ADGRB2 (a neural adhesion GPCR regulating synaptogenesis and angiogenesis) [25].

Assessment with Benchmarking Universal Single‐Copy Orthologs (BUSCO) using the mammalia_odb10 database (9,226 core genes) revealed a completeness of 98.6% for T2T‐RSC1.0, surpassing the 96.4% achieved with the previous RSC01 assembly (Figure S4B; Table S15). In addition, T2T‐RSC1.0 demonstrated an overall increase in the number of minimum unique k‐mers (MUK) across chromosomal regions compared with that obtained with RSC01 (Figure S4C,D). Overall, compared with RSC01, the T2T‐RSC1.0 assembly resolved an additional 8.7 Mb of previously unplaced bases, incorporated 129.19 Mb of PURs, and corrected 1,441,433 errors spanning 30.27 Mb (Table S11).

Collectively, these results confirm the consistency, reliability, and completeness of the T2T‐RSC1.0 assembly. This genome enables in‐depth exploration of previously inaccessible bat genomic sequences, structural features, gene expression patterns, and functional elements. Importantly, our assembly strategy, which combines HiFi and UL‐ONT long‐read sequencing with Hi‐C chromatin interaction data (Figure S5), achieves high contiguity and accuracy from a single sample without reliance on parental or trio genetic data. This approach offers a reference framework for genomic studies of non‐model organisms where pedigree samples are unavailable.

### 2.3 T2T‐RSC1.0 Advances Variant Calling and Evolutionary Inferences

To evaluate the performance of short‐read alignment and variant calling with the T2T‐RSC1.0 reference genome, we conducted population‐level whole‐genome resequencing of 52 *R. sinicus* individuals from eight geographic locations. Alignment of NGS reads to T2T‐RSC1.0 resulted in a lower error rate (0.948%) and a higher mapping rate (99.86%) than that achieved with the previous reference RSC01 (1.64% and 98.92%, respectively) (Figure 1F; Table S16). Using T2T‐RSC1.0, we identified a total of 7.02 × 10^6^ single‐nucleotide polymorphisms (SNPs) spanning 11 Gb across all populations. Approximately 3.01% (2.12 × 10^5^) of these SNPs were located within PURs. In addition, the use of T2T‐RSC1.0 improved the detection of insertions (INSs) and deletions (DELs), indicating enhanced accuracy in structural variant calling compared with that obtained with RSC01 (Figure S6A). RSC01 exhibits significantly more DELs than T2T in the satellite regions (Figure 1G), indicating more complete recovery of these repetitive sequences and higher assembly quality. In the PUR regions, DELs remained the predominant type of structural variation (Figure 1G; Tables S17–S19), suggesting high performance in the identification of structural variations. These results demonstrate that T2T‐RSC1.0 offers advantages as a reference genome in terms of read mapping, variant discovery, and the resolution of complex genomic regions.

*R. sinicus* is widely distributed and exhibits considerable genetic differentiation; studying the population genetics of this species may shed light on the mechanisms underlying species diversity and help prevent the cross‐species transmission of bat‐borne viruses. Using the T2T‐RSC1.0 reference genome, we performed population genomic analyses based on genome‐wide SNP variation and identified genome‐wide population structure corresponding to three genetic groups (Figure S6B). Phylogenetic reconstruction demonstrated that individuals from Fujian, Guangdong, Zhejiang, Anhui, Hunan, and Jiangxi form a Southeast Group (SE), whereas those from Sichuan and Yunnan comprise a Mid‐West Group (MW) and a Southwest Group (SW), respectively (Figure S6B). Both ADMIXTURE and principal component analysis (PCA), performed using either genome‐wide SNPs or Y‐chromosome SNPs, supported these three genetic clusters (Figure S6C,D).

Building upon previous population genetic studies [26, 27], our genome‐wide approach using the T2T‐RSC1.0 reference genome provides enhanced resolution for characterizing nuclear genetic structure and evolutionary diversification. We observed substantial genetic differentiation among the three groups, with the highest divergence between the SW and SE groups (weighted *F*_ST_ = 0.59), followed by the SW and MW (*F*_ST_ = 0.54) and SE and MW (*F*_ST_ = 0.44) groups (Figure S6E). Demographic history inferred via pairwise sequential Markovian coalescent (PSMC) analysis revealed divergent population trajectories among the groups. All three shared similar effective population sizes and dynamics until the Xixiabangma Glaciation (XG, approximately 80–120 thousand years ago (kya)) (Figure S6D). Subsequently, both the SE and MW groups experienced historical declines and a severe population bottleneck during the Penultimate Glaciation (PG, approximately 130 to 20 kya). In contrast, the SW group maintained stable population fluctuations. All groups underwent a bottleneck during the Last Glacial Maximum (LGM, approximately 20 kya) (Figure S6D).

Overall, the use of a complete reference genome enhances population genetic analyses by improving variant detection accuracy, improving read alignment in complex genomic regions, and supporting stronger inferences regarding population structure and variation discovery.

### 2.4 Architecture and Evolutionary Dynamics of the Bat Y Chromosome

The T2T‐RSC1.0 genome assembly exhibited a high repetitive content of 39.32%, surpassing that achieved with the previous RSC01 assembly (34.98%) (Table S11). Repetitive elements include LINEs (433.8 Mb, 20.27%), LTRs (138.2 Mb, 6.45%), and DNA transposons (109.2 Mb, 5.1%) (Tables S20,S21). The bat Y chromosome was dominated by repetitive sequences, which accounted for 79.19% of its sequence, with LINEs representing the major retrotransposon class (60.84%) (Figure 2A,B; Figure S7A). Compared with other mammals such as cattle [28], sheep [28], and humans [29], the bat Y shows a notable reduction in SINE abundance (0.18%) (Figure 2B; Table S20). We annotated 131 protein‐coding genes and five pseudogenes (4 copies of *GSDMC* and 1 copy of *LOC124900597*) on the Y chromosome (Table S22), establishing a genomic foundation for future studies on bat sex determination and male biology.

**FIGURE 2:**
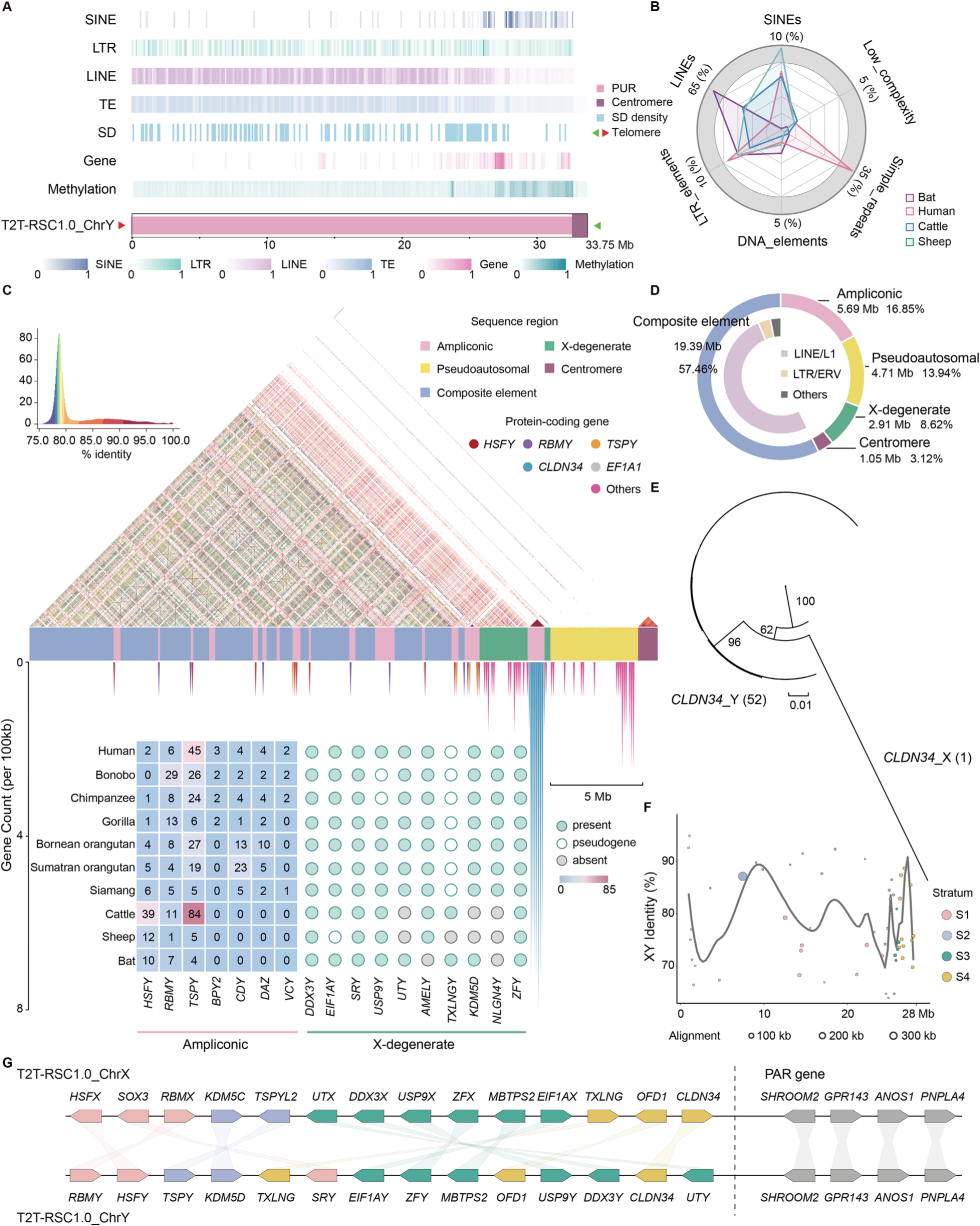
Architecture and evolutionary history of the bat Y chromosome. (A) Genomic features of T2T‐RSC1.0_ChrY. Using 100‐kb sliding windows, tracks depict the distribution of SINEs, LTRs, LINEs, other transposable elements (TEs), gene density, and DNA methylation along the Y chromosome. The Y chromosome is entirely classified as PUR. The color scale indicates relative feature density (0–1). The Y chromosome spans approximately 33.75 Mb, with telomeres at both ends (red and green triangles) and the centromere marked by a purple‐pink block. (B) Comparison of repeat composition between the bat Y chromosome and those of humans, sheep, and cattle showed a higher percentage of LINEs in the bat. (C) Triangular dot plot illustrating DNA sequence identity across the entire bat Y chromosome in a 10‐kb sliding window. The color gradient represents the percentage of sequence identity. Gene density, calculated in 100‐kb bins, highlights the distribution of protein‐coding genes along the Y chromosome. Copy number variation of genes within ampliconic and X‐degenerate regions among bats, humans, apes, cattle, and sheep. (D) Structural organization of the bat Y chromosome, showing the absence of the X‐transposed region in the bat. (E) Phylogenetic tree of *CLDN34* gene copies on the X and Y chromosomes in the bat. Node values represent Bootstrap support; the scale bar (0.01) indicates genetic distance. *CLDN34*_Y corresponds to 52 amplified copies on the Y chromosome, while *CLDN34*_X is a single copy located at the PAR boundary of the X chromosome. (F) Sequence divergence between the X and Y chromosomes reveals evolutionary strata. Each dot is a 100‐kb sliding window on the Y chromosome; values are derived from X–Y alignments. Distinct colors denote each evolutionary stratum; circle size scales with alignment length. (G) Distribution of four evolutionary strata on sex chromosomes of *R. sinicus*. Different colors indicate distinct evolutionary strata, whereas gray indicates gametologous pairs in the PAR, including the shared gene *CLDN34*.

By analyzing sequence similarity between the X and Y chromosomes, we identified a total length of 4.74 and 4.71 Mb of pseudoautosomal regions (PARs) in chrX and chrY, respectively, with 99.5% sequence identity (Figure 2C,D). The PARs are 1.52‐fold longer than those of humans [29] and contain 25 fewer protein‐coding genes than cattle and sheep PARs (Table S23). Key genes involved in germ cell development, fertilization, and reproduction, such as *PLCXD1*, *SHOX*, and *GTPBP6*, were absent from the bat Y chromosome, indicating lineage‐specific differences in PAR gene content compared with other mammals [28]. The PARs were found to possess two copies of *ASMTL* and *CSF2RA* (Table S23); these genes regulate oxidative stress responses, DNA damage repair, immune regulation, and the inflammatory response [30, 31].

In addition to PARs, the male‐specific Y chromosome (MSY) contains the candidate centromeric (1.05 Mb), X‐degenerate (2.91 Mb), ampliconic (5.69 Mb), and composite element (19.39 Mb) regions (Figure 2D). We did not identify any X‐transposed regions in the bat Y chromosome based on methods of both species‐specific and sequence class annotations (Figure 2D), consistent with findings in apes [32], cattle [28], and sheep [28]. We identified a 1.05‐Mb candidate centromeric region enriched in satellite repeats between 32.70 and 33.75 Mb, a location consistent with a telocentric organization of the Y chromosome (Figure 2A,B; Figure S7B). This candidate centromeric region exhibited heterochromatic features based on ATAC‐seq and CUT&Tag signal patterns (Figure S7C). Composite elements spanned 19.39 Mb, accounting for 57.46% of the Y chromosome and representing a major structural component of the MSY (57.46%), thus constituting a major part of the Y chromosome (Figure 2C,D). Specifically, LINE/L1 serves as the dominant repetitive element (88.71%) (Table S20). LTR elements rank as the second‐largest type, with their subfamilies (LTR/ERVK, LTR/ERVL‐MaLR, LTR/ERVL, and LTR/ERV1) accounting for 2.06%, 1.52%, 1.32%, and 1.15%, respectively (Figure 2D; Table S20).

The bat Y chromosome retains few X‐degenerate sequences, with a total length of 2.91 Mb (Figure 2D). The X‐degenerate region originates from the autosomal ancestor of mammalian sex chromosomes and retains genes with a certain level of homology to the X chromosome, including 16 protein‐coding genes (*HSFY1*: 1, *Prss55*: 1, *TSPY2*: 2, *KDM5D*: 1, *TXLNG*: 3, *EIF1AY*: 1, *ZFY*: 1, *MBTPS2*: 1, *OFD1Y*: 1, *USP9Y*: 1, *DDX3Y*: 1, *UTY*: 1, and *SRY*: 1) and four pseudogene copies of *GSDMC* (Figure 2C; Table S24).

The ampliconic regions were found to measure 5.69 Mb in length, comprising 16.85% of the Y chromosome (Figure 2D). Among all sequence classes on the Y chromosome, the ampliconic regions exhibited the highest gene density, and the genes in this region were closely related to spermatogenesis [33]. We identified five species‐specific protein‐coding gene families, including two copies of *EF1A1*; three copies of *TSPY*; seven copies of *RBMY*; 11 copies of *HSFY* and 52 copies of *CLDN34*, respectively (Figure 2C; Table S25). Furthermore, transcriptional activity was detected for members of all five gene families (Figure S7D; Table S26). A further analysis of gene arrangement revealed that the *HSFY and RBMY* genes in the bat ampliconic region are mainly arranged in tandem arrays (Table S22). This feature is consistent with the tandem arrangement pattern of homologous genes in the cattle Y chromosome ampliconic region (the *HSFY*, *RBMY*, *PRAME*, and *TSPY* genes are tandemly distributed), which is likely highly related to efficient gene replication and coordinated expression [28]. No pseudogenes were annotated in the bat ampliconic region, contrasting with observations in cattle (50 pseudogenes) and sheep (127 pseudogenes) and highlighting lineage‐specific differences in the structural organization of mammalian Y chromosomes [28].

We observed a marked expansion of the *CLDN34* gene family, with 52 copies identified in *R. sinicus* (Figure 2C; Figure S7D). In contrast, the X chromosome harbors a single *CLDN34* ortholog, located at the PAR boundary, a genomic configuration that is consistent with other mammalian lineages, such as *Nycticebus pygmaeus* [34] and *Daubentonia madagascariensis* [34]. Approximately 36.5% of Y‐linked copies showed transcriptional activity, with the expressed copies detected specifically in the testis, whereas the single X‐linked copy displays broad expression across diverse tissues, including wing membrane, lung, and spleen. Molecular evolutionary analyses uncovered strong divergence, with an elevated *ω* of 2.60943 between the X–Y‐linked loci. Phylogenetic reconstruction showed that the X‐linked copy was clearly separated from a monophyletic cluster of Y‐linked copies, consistent with a recent and rapid expansion of the Y‐linked copies (Figure 2E). The dramatic recruitment and expansion of *CLDN34* on the Y chromosome, together with pronounced sequence divergence and strict testis‐specific expression of the transcriptionally active Y‐linked copies, suggest lineage‐specific functional specialization in male reproductive processes. This pattern highlights *CLDN34* as a candidate Y‐linked gene family potentially involved in reproductive or spermatogenic processes.

To further reveal the sex chromosomes' divergence from each other, we evaluated the evolutionary strata pattern by assessing the sequence similarity between the X and Y chromosomes. We found that sequence similarity between the sex chromosomes increased from older to younger strata (Figure 2F). The synonymous substitution rate (*dS*) measures the evolutionary age of X–Y gene pairs [33]. Based on mapping and phylogenetic analyses, we speculated that there are four evolutionary strata (S1–S4, oldest to youngest) in the bat sex chromosomes (Figure 2F). Fourteen MSY genes are gametologs of X‐chromosomal genes, and the pairwise *dS* of these X–Y gametologs declines with greater distance from the *SOX3* gene on the X chromosome (Figure 2F,G; Table S27). The gametologous gene pair in S3 was found to exhibit lower *dS* values, with 0.216311 as the minimum value, consistent with regions belonging to a young evolutionary stratum (Table S27). The relatively low *dS* values and high similarity of these gametologous pairs are consistent with a more recent cessation of X–Y recombination. The genes in S3 are all located in the X‐degenerate region (Figure 2F; Table S27), including four Y‐linked genes critical for male sex determination and spermatogenesis: *ZFY*, a transcription factor that initiates the sex determination pathway [35]; *DDX3Y*, which regulates RNA unwinding and translation initiation [36]; *USP9Y*, a ubiquitin‐specific protease that maintains protein stability [37]; and *UTY*, which exhibits histone demethylase activity and participates in immune regulation [38]. These findings offer valuable insights into the evolution of sex chromosomes in Chiroptera.

### 2.5 Improved Gene Annotation Reveals Trait‐Associated Genomic Features in a Coronavirus Reservoir Host

Through a combination of full‐length transcript (Iso‐seq) data, RNA‐seq, and integrated ab initio and homology‐based gene prediction, we annotated 20328 protein‐coding genes, representing 99.2% of complete conserved orthologs within mammals (Table S28). In our functional annotation, 99.63% and 98.80% of the annotated genes were assigned entries in the NR and Swiss‐Prot databases, respectively (Table S29).

A total of 848 genes located in PURs were newly identified in T2T‐RSC1.0, regions absent in RSC01 (Table S14). Functional enrichment analysis revealed that genes in PURs are highly pleiotropic, spanning diverse biological processes including embryo development, DNA damage checkpoint signaling, Hippo signaling, stress responses, and immunity (Figure 3A; Table S30). Among these, 20 of 848 genes were associated with immune or antiviral functions (Figure 3B; Table S30). Interestingly, the pre‐pro‐defensin alpha 5 (DEFA*5*) gene (known to mediate epithelial antiviral defenses [39]) that has previously been thought to be lost in bats [40] was found to harbor an intact open reading frame in *R. sinicus*. We observed *DEFA5* expression in stomach and spleen tissues, and the transcriptional activity was also confirmed in vitro in transfected HEK293T cells (Figure S8).

**FIGURE 3:**
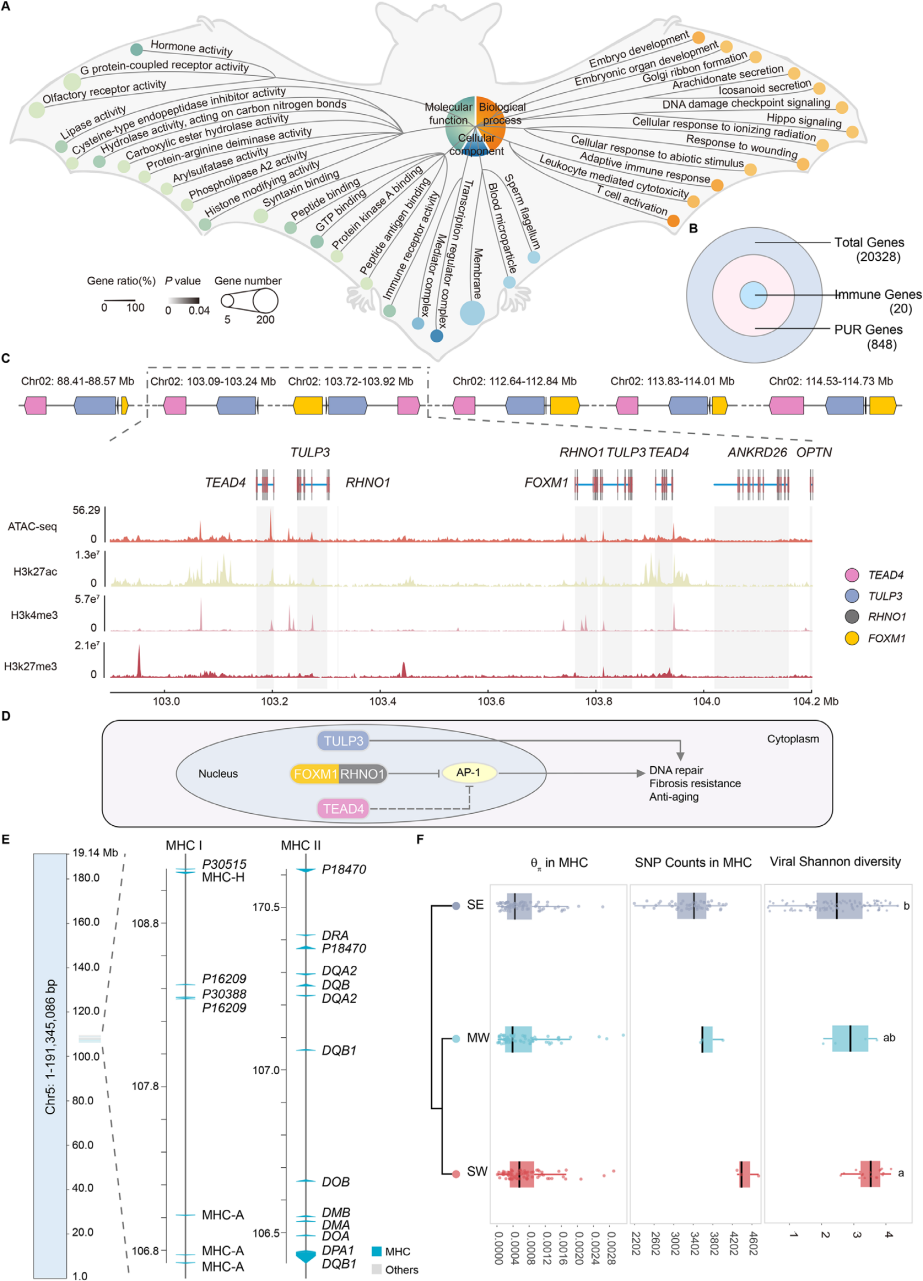
Functional characteristics of PURs and MHC genomic architecture in *R. sinicus*. (A) Functional enrichment analysis of genes located in PURs. Gene Ontology (GO) enrichment was conducted using Fisher's two‐sided exact test, with *P*‐values adjusted for multiple testing using the Benjamini–Hochberg method. Significantly enriched GO terms are shown for Biological Process (orange), Molecular Function (green), and Cellular Component (blue). (B) Schematic diagram illustrating the overlap among all annotated genes (*n* = 20328), genes located within PURs (*n* = 848), and immune‐related genes (*n* = 20). (C) Genomic organization and epigenetic landscape of the tandemly repeated gene cluster in the PURs on chromosome 2. Gene loci (*TEAD4*, *TULP3*, *RHNO1*, *FOXM1*) are indicated below (chr02: 102.9 to 104.2 Mb), with corresponding epigenetic tracks displayed, including ATAC‐seq (orange), H3K27ac (yellowish‐brown), H3K4me3 (pink), and H3K27me3 (red). (D) Schematic model of the anti‐senescence regulatory module involving *FOXM1*, *TEAD4*, *RHNO1*, and *TULP3*. (E) Genomic organization of MHC class I and class II regions in *R. sinicus*, with MHC class I and class II genes shown in blue and non‐MHC genes in gray. (F) Population‐level polymorphism of the MHC region and viral diversity across three genetic lineages (SE, MW and SW). Plots include SNP counts, MHC nucleotide diversity, and viral Shannon diversity, based on 52 *R. sinicus* individuals with paired genomic and viral screening data. Viral Shannon diversity between lineages was compared by Hutcheson’s *t*‐test.

Notably, we identified a tandemly repeated gene array within PURs, comprising *TEAD4*, *TULP3*, *RHNO1*, and *FOXM1* (Figure 3C; Table S14). Among these, *RHNO1* and *TEAD4* are primarily associated with Hippo signaling, embryonic development, embryonic organ development, and cellular responses to abiotic stimuli (Table S14). These genes have been shown to coordinately regulate senescence‐associated phenotypes, including cell‐cycle arrest and replication stress [41]. Mechanistically, the coordinate transcription of *FOXM1*, *TEAD4*, and *RHNO1* constitutes a coherent anti‐senescence regulatory module. *FOXM1* suppresses the senescence‐associated pioneer factor AP‐1 to restore youthful chromatin accessibility, *TEAD4* promotes cellular proliferation through Hippo signaling, and *RHNO1* alleviates replication stress by activating the ATR‐mediated DNA damage response [41, 42]. *TULP3* further inhibits fibrotic aging by maintaining ciliary integrity and restraining TGF‐β signaling [41, 43]. We also found that this gene cluster of *TEAD4*, *TULP3*, and *RHNO1* exhibits lineage‐specific expansion in *R. sinicus* with high expression levels, especially in kidney tissue. The co‐localization of H3K4me3 and H3K27ac peaks also indicates transcriptional regulatory activity in this region (Figure 3C,D). The increased gene dosage suggests a potentially adaptive role in maintaining cellular homeostasis and mitigating replication stress, although the precise biological consequences remain to be experimentally validated. Similarly, the *KNG1* and *HRG* genes are likewise exclusively present within PURs, where they form a structurally continuous tandemly repeated array and are highly expressed in the liver. Functional enrichment analysis revealed that both gene sets are significantly associated with key regulatory pathways, including hemostasis, blood coagulation, and wound healing (Figure 3A; Table S30), suggesting that they may act cooperatively or complementarily in maintaining blood homeostasis and facilitating tissue repair.

In summary, T2T‐RSC1.0 provides a comprehensive gene catalog for the horseshoe bat, establishing a foundational resource for exploring mechanisms of bat–virus immune adaptation and host–virus interactions.

### 2.6 Association Between Host MHC Diversity and Viral Diversity Among Three Populations

The complete genome also enabled the precise reconstruction of complex immunogenomic regions. Major histocompatibility complex (MHC)‐encoded molecules govern immune responses by presenting antigenic peptides to T cells [44, 45]. Leveraging a gap‐free genome, multi‐tissue and full‐length transcriptomes, and comparative annotations from human, mouse, rat, *Pteropus vampyrus*, and *Pteropus alecto* (*P. alecto*), we fully assembled a 3.53 Mb MHC cluster in *R. sinicus*, including class I genes MHC‐H and MHC‐A, and class II genes *DQB1*, *DPA1*, *DOA*, *DMA*, *DMB*, *DOB*, *DQA2*, *DQB*, and *DRA* (Figure 3E; Table S31). The MHC‐A locus exhibits copy number expansion, reminiscent of Pongo [16], while class I is 2.6‐fold larger than in *P. alecto* [46] and class II remains similar in size [47], reflecting distinct selective pressures on intracellular versus extracellular pathogen defense. *R. sinicus* retains a functional *DPA1* and shows duplications of *DQB1* and *DQA2*, enhancing antigen presentation diversity, while no class II genes are found outside the canonical region (Figure 3E; Table S31), in contrast to *P. alecto* [46, 47]. These findings reveal lineage‐specific plasticity of bat MHC shaped by pathogen‐driven selection and underscore the power of T2T‐RSC1.0 for resolving complex immunogenomic architecture.

Population‐level analyses revealed that MHC polymorphism mirrored host phylogeography: individuals from Fujian, Guangdong, Zhejiang, Anhui, Hunan, and Jiangxi clustered as a Southeast Group (SE), while those from Sichuan and Yunnan formed Mid‐West (MW) and Southwest (SW) groups, respectively (Figure S6). The SW population exhibited the highest nucleotide diversity (*π*) and SNP counts (*n* = 4470) in the MHC region, far exceeding SE and MW (Figure 3F; Table S32). Concordantly, viral α‐diversity was significantly elevated in the SW group compared to the SE group (*P* < 0.01; Figure 3F; Table S32), aligning with host genetic patterns and suggesting that differences in local ecological conditions, host genetic background, and viral exposure may contribute to the observed variation among populations. This pattern is consistent with population‐level differences in host MHC diversity. High MHC polymorphism may contribute to broad antigen recognition, which in turn imposes selective pressure on viral communities. These findings suggest potential interactions between host immunogenomic variation and viral diversity during long‐term host–virus interactions.

### 2.7 Comparative Genomics Reveals Evolutionary Immune Adaptation in Horseshoe Bats

High‐quality genome assemblies of horseshoe bats provide a foundation for investigating the evolutionary dynamics of immune pathways. We conducted a comparative genomic analysis leveraging currently available high‐quality bat genomes and those of model species, totaling 15 mammalian genomes. We identified 9,709 single‐copy orthologous genes among the 15 species (Table S33). We reconstructed a genome‐wide phylogenetic tree using the maximum likelihood (ML) method and obtained 100% bootstrap support at all nodes (Figure 4A), consistent with the topology of the recent Laurasiatherian phylogenies [7]. The chiropteran species diverged into two major clades approximately 67.89 million years ago (Mya), corresponding to the Yinpterochiroptera and Yangochiroptera suborders. Within *Rhinolophus*, *Rhinolophus* ferrumequinum (*R. ferrumequinum*) represents the basal lineage, having diverged around 16.32 Mya, while *R. sinicus* split approximately 8.6 Mya (Figure 4A).

**FIGURE 4:**
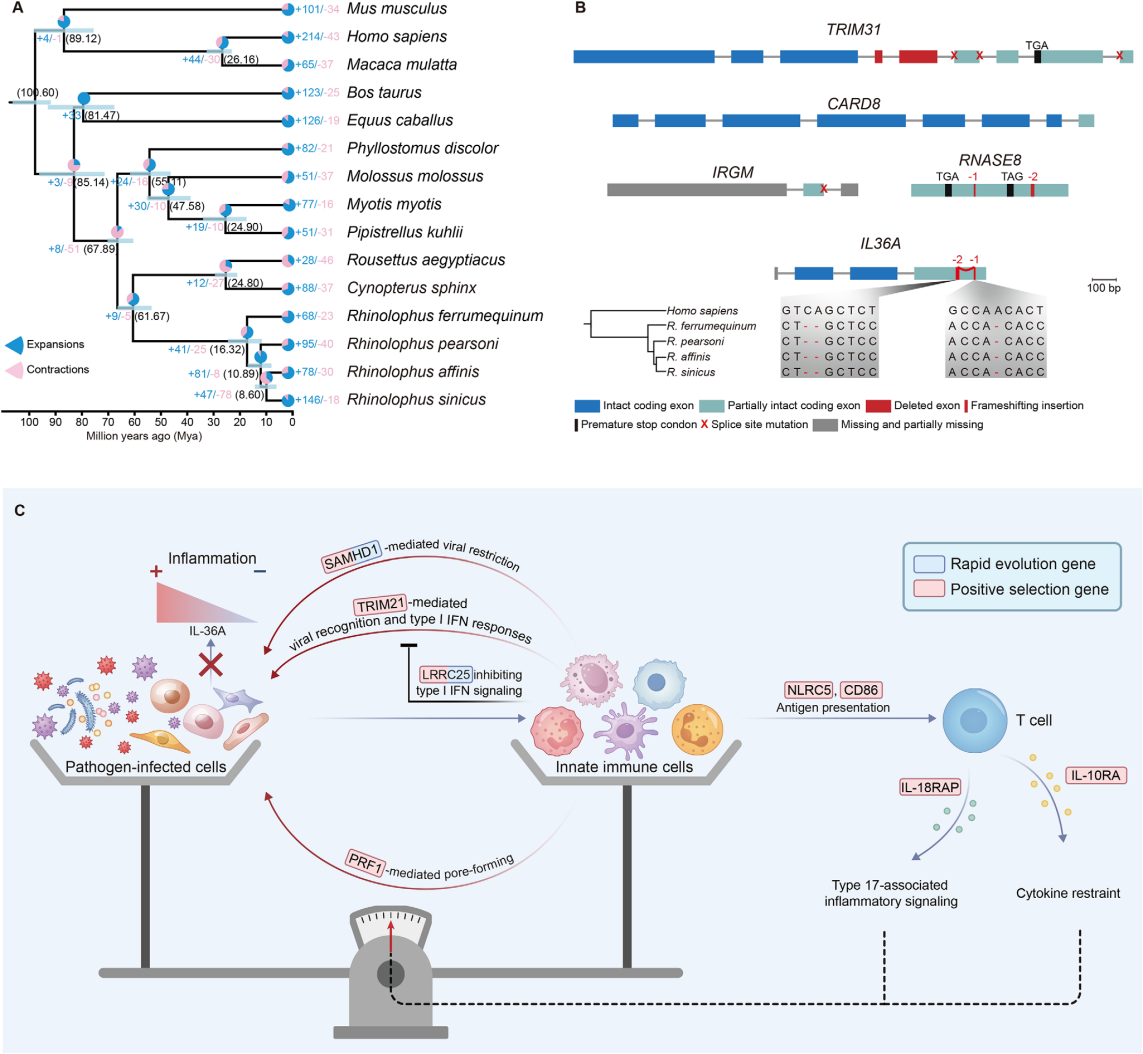
Evolutionary patterns of immune regulation in *Rhinolophus*. (A) Summary of Laurasiatherian phylogeny according to 9,709 single‐copy orthologous genes. All nodes received 100% bootstrap support. Gene family evolution and divergence time were indicated. (B) Diagram showing the pseudogenization‌ of inflammation‐related genes. *IL‐36A* is pseudogenized in all examined *Rhinolophus* species. (C) Proposed model of evolutionary remodeling of immune regulatory networks in *Rhinolophus* bats. The model summarizes representative immune‐associated genes showing signatures of positive selection (pink) or rapid evolution (blue), including genes involved in antiviral defense, antigen presentation, cytokine signaling, and immune regulation. This framework highlights evolutionary changes in antiviral defense‐related mechanisms and regulatory pathways that may contribute to fine‐tuning immune activation and inflammatory responses during long‐term host–virus interactions.

Compared with the other 14 species, *R. sinicus* was found to exhibit 18 significantly contracted and 146 significantly expanded gene families, among which 25 were found to be specifically expanded in *R. sinicus* (Figure 4A; Table S34). These 146 significantly expanded genes participate in pathways such as hydrolase activity, NF‐κB signaling, and cell cycle regulation (*P* < 0.001; Table S35). For example, *RPS3* contributes to NF‐κB signaling by modulating NF‐κB DNA‐binding and transcriptional activity [48, 49]. *EEF1A1* has been implicated in mediating the secretion of proinflammatory cytokines (including IL‐18) through NF‐κB‐dependent mechanisms [50]. *HSP90AB1* promotes the epithelial‐mesenchymal transition (EMT) and metastasis in several cancers, acting in concert with elongation factors to enhance pro‐EMT signaling [51]. *NPM1* has been reported to suppress *NLRC5* transcription and thereby inhibit NLRC5‐dependent MHC‐I antigen‐presentation pathways [52]. Together, these lineage‐specific expansions highlight evolutionary remodeling of immune‐related pathways and cellular regulatory processes in *R. sinicus*.

In *R. sinicus*, we identified a total of 1119 pseudogenes using TOGA. Functional enrichment analyses showed that these pseudogenes are significantly overrepresented in pathways such as the negative regulation of NLRP3 inflammasome complex assembly (e.g., *TRIM31*, *CARD8*, *IRGM*; adjusted *P* < 0.01) and the defense response to Gram‐negative bacteria (e.g., *RNASE8*; adjusted *P* < 0.01) (Figure 4B; Table S36). Among these pseudogenes, the proinflammatory cytokine *IL‐36A* has been found to be pseudogenized in all examined *Rhinolophus* species, with Exon 1 entirely missing and Exon 4 harboring two frameshift mutations; this pseudogenization represents a lineage‐wide evolutionary alteration of inflammatory regulation that may contribute to attenuation of inflammatory signaling [7] (Figure 4B).

We conducted genome‐wide selection analyses using PAML based on 9,709 single‐copy orthologous genes across 15 mammalian species. In the *R. sinicus* lineage, we identified 85 positively selected genes (PSGs) and 16 rapidly evolving genes (REGs) using a multiple‐testing‐corrected threshold (*q* < 0.05) (Tables S37–S38). Several immune‐associated genes exhibited lineage‐specific evolutionary signatures, suggesting selective remodeling of immune‐related pathways. *NLRC5*, a transcriptional regulator of MHC‐I antigen presentation, underwent positive selection, suggesting evolutionary modification of antigen presentation pathways [53]. *PRF1* (perforin‐1), a pore‐forming effector molecule released by cytotoxic lymphocytes, showed signatures of both positive selection and rapid evolution, potentially influencing cytotoxic immune responses [54]. In addition, *IL1RAP*, an accessory component of IL‐1 family receptor signaling, was positively selected, suggesting evolutionary tuning of cytokine signaling pathways [55]. Other genes involved in immune regulation and cellular homeostasis, including *BECN1*, further support lineage‐specific remodeling of immune regulatory pathways [56].

Beyond species‐specific evolutionary changes, we further investigated adaptive evolution across the Rhinolophus genus. We identified 236 PSGs and 21 REGs after multiple‐testing correction (*q* < 0.05) (Figure 4C; Tables S39, S40). Evolutionary changes in antiviral defense pathways involved multiple layers of host–virus interaction (Figure 4C). *TRIM21*, an intracellular antibody‐dependent viral recognition factor involved in antiviral signaling and type I interferon responses, showed signatures of positive selection [57]. *SAMHD1*, exhibiting signatures of both positive selection and rapid evolution, encodes a nucleoside triphosphohydrolase that restricts viral replication by reducing intracellular deoxynucleotide availability [58]. In addition, *PRF1* (perforin‐1), a pore‐forming cytotoxic effector molecule released by NK cells and cytotoxic lymphocytes, represented a recurrent target of selection across evolutionary scales [54], from the *R. sinicus* lineage to the *Rhinolophus* genus, highlighting adaptive modification of host effector mechanisms involved in elimination of infected cells.

In parallel, evolutionary signatures were detected in regulatory pathways that shape immune activation thresholds. *LRRC25*, a regulator of interferon signaling, may contribute to fine‐tuning type I interferon responses and maintaining immune homeostasis [59]. Genes associated with antigen presentation and adaptive immune regulation, including *NLRC5* and *CD86*, suggest evolutionary modification of pathways connecting innate immune recognition with T‐cell activation [60, 61]. Notably, *NLRC5* showed evolutionary signatures at both species and genus levels, suggesting recurrent selection on antigen presentation pathways during horseshoe bat evolution. Furthermore, *IL18RAP* and *IL10RA* represent evolutionary changes in cytokine signaling networks associated with inflammatory regulation and immune restraint [62, 63].

Collectively, these comparative genomic analyses reveal a coordinated evolutionary remodeling of immune regulatory networks in *Rhinolophus* bats, characterized by the integration of antiviral defense programs with regulatory pathways that fine‐tune immune activation and inflammatory responses.

### 2.8 Integration of the Splenic Atlas and Immune Stimulation Assays Reveals Distinct Regulation of Interferon‐Associated and Inflammatory Responses in Chinese Horseshoe Bats

To delineate the cellular organization and transcriptional states associated with immune regulation in Chinese horseshoe bats, we constructed a transcriptomic cell atlas of the spleen, a key immune organ, from two adult *R. sinicus* individuals using single‐cell RNA sequencing (scRNA‐seq). Following quality control, we obtained high‐quality transcriptomic profiles from 26,102 cells, with a median gene detection range of 1014 to 1199 genes per cell (Table S41).

We identified immune cell populations based on conserved marker genes informed by published scRNA‐seq datasets from human and mouse spleens and Egyptian rousette bat blood [64, 65, 66]. The initial annotation revealed 14 major cell types, including T cells, B cells, macrophages, monocytes, neutrophils, NK cells, dendritic cells, mast cells, myeloid cells, and plasma cells (Figure 5A). We further conducted a cross‐species comparison of marker gene expression among the shared and abundant cell types, namely T cells, B cells, plasma cells, macrophages, and neutrophils, across humans, mice, and horseshoe bats (Figure 5B). In the identification of T cell populations in the horseshoe bat, we observed that T cells were characterized by the expression of CD3d, consistent with markers used in humans and mice [65, 66]. In addition, bat T cells expressed CTLA4, a T‐cell surface immune checkpoint molecule that downregulates T‐cell‐mediated immune responses by competitively inhibiting CD28 co‐stimulatory signaling [67] (Figure 5B). The B cells expressed CD19 and CD79a along with MS4A1 (CD20) and CD74 (Figure 5B). Plasma B cells were marked by MZB1 expression, a pattern consistent with that observed in humans and mice [65, 66]; these cells were additionally characterized by IGHM and IGLL5 expression (Figure 5B). Bat macrophages expressed CD68 and CD163, as well as LYZ, which encodes lysozyme, a key effector of innate immunity [68], and G0S2, which is involved in immune‐cell metabolic regulation [69] (Figure 5B). Neutrophils were specifically marked by F13A1 (Coagulation Factor XIII A Chain) (Figure 5B), which has been implicated in regulating macrophage function and inflammatory responses [70], suggesting a potential role in modulating infection response and tissue repair.

**FIGURE 5:**
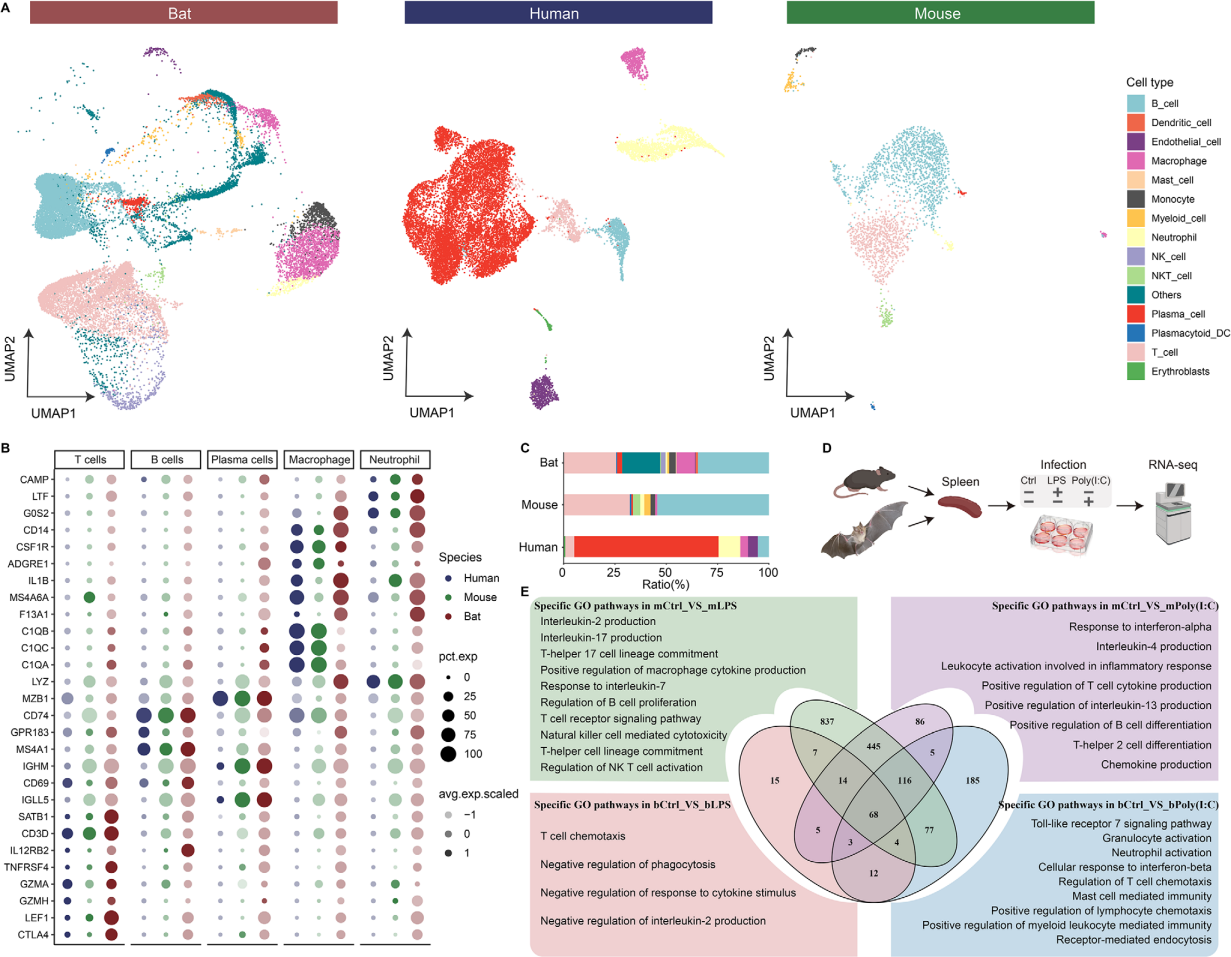
Integrated splenic cell atlas and immune stimulation assays reveal species‐specific immune responses in Chinese horseshoe bats. (A) UMAP visualization of all cells in the spleens of bats, humans, and mice. The color annotation indicates each annotated cell type. (B) Dot plots showing the expression of key marker genes (*x*‐axis) of major cell types (*y*‐axis) in bats, humans, and mice. (C) Comparison of the ratio of cell types among bats, humans, and mice. The color of each cell type is the same as that in A. (D) Schematic overview of LPS and poly(I:C) stimulation assays using splenic cells from bats and mice. (E) Venn diagram of GO enrichment of the differentially expressed genes between stimulated and control groups in bats and mice. The specific GO pathways in each comparison pair are highlighted.

We compared our manually annotated cell types with automated annotations derived from human and mouse single‐cell reference databases (human: Azimuth; mouse), and observed differences in cell‐type proportions across species. The horseshoe bat exhibited a higher proportion of innate immune cells, including macrophages (8.97%), monocytes (3.24%), NK cells (2.17%), and dendritic cells (1.09%), and relatively fewer T and B cells compared with humans and mice (Figure 5C). This altered cellular composition reveals species‐specific organization of immune cell populations in Chinese horseshoe bats.

To investigate how immune regulatory features are manifested at the cellular and transcriptional levels, we compared the splenic responses between mice and bats following LPS or Poly(I:C) stimulation (Figure 5D). In mice, LPS stimulation induced canonical inflammatory signatures, including cytokine signaling responses involving IL‐2, IL‐7, and IL‐17, macrophage activation, NK cell responses, and cytotoxicity programs (Figure 5E; Figure S9A). In contrast, bats exhibited reduced inflammatory transcriptional signatures, including IL‐2‐associated responses, together with altered phagocytosis‐related innate immune pathways (Figure 5E; Figure S9B).

Following poly(I:C) stimulation, mice exhibited increased expression of cytokine‐associated inflammatory responses, including IL‐4, IL‐13, and IFN‐α signaling pathways (Figure 5E; Figure S9A). In contrast, *R. sinicus* displayed activation of TLR7 signaling, granulocyte responses, and IFN‐α production, indicating species‐specific regulation of interferon‐mediated antiviral responses (Figure 5E; Figure S9B). Together, these analyses reveal that immune regulation in Chinese horseshoe bats is characterized by coordinated modulation of immune activation states rather than simple enhancement of immune responses.

## 3 Discussion

This study presents a high‐quality genomic framework for investigating genome evolution, population differentiation, and immune adaptation in the Chinese horseshoe bat (*R. sinicus*). The pedigree‐free, gap‐free chromosome‐level assembly generated from a single individual provides a broadly applicable reference resource for non‐model organisms where pedigree information is unavailable. Complete resolution of the X and Y chromosomes enables investigation of sex chromosome evolution in bats, including a four‐strata evolutionary pattern. Genome‐wide population analyses characterized host–virus genetic differentiation within *R. sinicus*, while comparative genomic and multi‐omics analyses provide insights into immune regulation and host–virus interactions in horseshoe bats. These findings establish a foundation for exploring how genomic evolution shapes adaptive traits in bats.

The male‐specific Y chromosome plays a critical role in sex determination and male fertility [33]. However, relatively complete Y chromosome assemblies are limited to only a few species due to their repetitive nature and incomplete reference assemblies [71]. Here, we present gapless assemblies of the X and Y chromosomes in *R. sinicus*, enabling direct characterization of previously unresolved features of chiropteran sex chromosome evolution and providing new insights into the mechanisms underlying sex chromosome diversification. The bat Y chromosome is characterized by a high abundance of LINE repeat elements and a degradation of an X‐transposed region, which is associated with reduced homologous recombination with the X chromosome and may contribute to accelerated evolutionary divergence. Similar patterns of repeat accumulation and recombination suppression have been reported across mammalian Y chromosomes, suggesting that the bat Y chromosome follows conserved principles of sex chromosome degeneration while exhibiting lineage‐specific evolutionary trajectories.

Previous studies in *Pteropus* and *Rousettus* bats have suggested that altered interferon signaling, attenuated inflammatory responses, and immune regulation contribute to viral tolerance [72, 73]. However, whether these observations represent isolated pathway modifications or coordinated evolutionary remodeling of immune regulation remains unresolved, particularly in Rhinolophid bats that serve as natural reservoirs of SARS‐related coronaviruses. Our MHC analyses revealed population‐level associations between host immunogenomic variation and viral diversity. These patterns are consistent with the possibility that long‐term host–virus interactions, including potential co‐evolutionary processes, may contribute to shaping immunogenomic diversity in bats. Our multi‐layered genomic and functional analyses revealed that immune adaptation in horseshoe bats is shaped by coordinated remodeling of antiviral defense and inflammatory control rather than broad activation of immune pathways. These findings provide an evolutionary framework for understanding how natural reservoir hosts balance pathogen defense with immune restraint during long‐term host–virus interactions.

This study has several limitations that should be considered. First, although the population genomic analysis encompassed a large proportion of the known geographic distribution of *R. sinicus*, the sample size remained limited. Each identified genetic population was represented by at least three biological replicates, providing support for the inferred population structure. However, larger population sampling will be required to further characterize genetic diversity and demographic history. Second, our conclusions regarding immune adaptation, potential viral tolerance‐associated features, and host–virus interactions are primarily based on comparative genomics, transcriptomic profiling, and in vitro immune stimulation assays. Although these approaches identified candidate genes, evolutionary signatures, and immune regulatory patterns, they do not directly establish causal mechanisms underlying viral tolerance or host–virus coexistence. In addition, although cross‐species comparisons of immune cell composition provide valuable insights into conserved and divergent immune features, differences in experimental platforms and annotation strategies among species should be considered when interpreting relative cell‐type proportions. Future studies using in vivo infection models and functional validation will be required to determine the physiological relevance of these genomic and immunological features.

## 4 Conclusion

In summary, this study provides a complete T2T genome for the horseshoe bat *R. sinicus*, a key natural reservoir of SARS‐related coronaviruses. This resource enables the decoding of genomic and cellular blueprints underlying bat‐specific immune responses, adaptive evolution, and host–virus interactions. Our findings provide evolutionary insights into how natural reservoir hosts maintain immune homeostasis during long‐term interactions with diverse viruses, offering translational insights for managing infections and immunopathology in humans.

## 5 Methods

### 5.1 Source Organism

Adult *R. sinicus* individuals used in this study were collected from different sampling locations in China (Tables S1, S16). The reference individual used for T2T genome assembly was collected from Jiangxi Province, China (117.25°E, 27.88°N).

Muscle tissue collected from this individual was used for extraction of high‐molecular‐weight genomic DNA for short‐read sequencing, PacBio HiFi sequencing, Oxford Nanopore ultra‐long sequencing, and Hi‐C sequencing.

Additional individuals from Jiangxi Province were used for multi‐omics analyses. Samples used for chromatin accessibility and histone modification profiling (ATAC‐seq and CUT&Tag) were collected from individuals captured in Jiangxi Province, China (115.76°E, 28.80°N). For transcriptomic analyses, total RNA was extracted from eight tissues (wing membrane, heart, muscle, stomach, kidney, spleen, liver, and lung) collected from six adult *R. sinicus* individuals (four males and two females) captured in Jiangxi Province, China (117.25°E, 27.88°N). Intestinal tissues used for metavirome sequencing and individuals used for whole‐genome resequencing were sampled from the same populations, and the WGS panel included 52 individuals (Table S6).

### 5.2 Tissue and Cell Preparation From Bat

Adult male Chinese horseshoe bats (*R. sinicus*) were captured and transferred to the lab. Animals were anesthetized with pentobarbital sodium (75 mg/kg body weight) prior to tissue collection. Tissues including muscle, heart, liver, lung, spleen, stomach, kidney, testis, and wing membrane were harvested for subsequent analyses.

Spleens from bats or mice were rapidly dissected and rinsed in ice‐cold Dulbecco's phosphate‐buffered saline (DPBS). The tissues were gently dissociated through a 70‐µm cell strainer using sterile plungers. Cell suspensions were collected into 50‐mL centrifuge tubes and centrifuged at 300 × g for 5 min at 4°C. Pellets were resuspended in 3 mL red blood cell lysis buffer and incubated on ice for 1 min. The lysis reaction was quenched by adding 20 mL DPBS supplemented with 2 mm EDTA and 0.5% bovine serum albumin (BSA).

Cells were centrifuged again at 300 × g for 5 min at 4°C, washed twice with DPBS, and counted using a hemocytometer following trypan Blue staining. Cells were cultured in complete RPMI‐1640 medium supplemented with 10% heat‐inactivated fetal bovine serum, 100 U/mL penicillin, and 100 mg/mL streptomycin.

### 5.3 PacBio HiFi Sequencing

SMRTbell libraries were prepared from ultra‐high‐molecular‐weight genomic DNA extracted from muscle tissue of an adult *R. sinicus* individual. DNA was sheared and size‐selected to approximately 25 kb using the PacBio SMRTbell Express Template Prep Kit, following the manufacturer's instructions. Four SMRTbell libraries were sequenced on the PacBio Sequel II platform, and raw data were processed using SMRT Link v12.0.

In total, ∼121.28 Gb of HiFi sequencing data were generated, comprising 7,130,x084 reads with a mean read length of 17,009 bp, a maximum read length of 57,332 bp, and a read N50 of 17,220 bp. Reads longer than 10, 20, and 40 kb accounted for 97.94%, 20.39%, and 0.02% of the total reads, respectively.

### 5.4 Ultra‐Long ONT Sequencing

Ultra‐long libraries (∼150 kb) were prepared from ultra‐high‐molecular‐weight genomic DNA extracted from muscle tissue of the reference *R. sinicus* individual used for T2T genome assembly. For each library, approximately 8–10 mg of genomic DNA was size‐selected (>50 kb) using the SageHLS HMW Library System and processed with the Ligation Sequencing Kit 1D following the manufacturer's protocol. Approximately 800 ng of DNA library was loaded per run and sequenced on the Oxford Nanopore PromethION 48 platform at the Genome Center of GrandOmics. Raw fast5 files were basecalled into fastq format using Guppy (sup mode) [74].

The total sequencing output was ∼247.08 Gb, comprising 4,868,905 reads with a mean read length of 50,746 bp, a maximum read length of 919,570 bp, and a read N50 of 116,464 bp. Reads longer than 10, 20, and 40 kb accounted for 69.53%, 55.57%, and 38.19% of the total reads, respectively.

### 5.5 Short‐Read Sequencing

Ultra‐high molecular weight genomic DNA was extracted from bat muscle tissue and used to construct paired‐end 150 bp (PE150) libraries for whole‐genome sequencing (WGS) on the DNBSEQ‐T7 platform.

Base accuracy exceeded 99%, and raw sequencing reads were quality‐checked using fastp (v0.23.4) [75] with default parameters. A total of 126.03 Gb of raw data, comprising 840,174,226 reads, were generated. After filtering low‐quality reads, 116.99 Gb of clean data (840,090,–308 reads) were retained, with Q30 and Q20 scores of 97.69% and 92.45%, respectively, and a GC content of 40.76%.

### 5.6 Hi‐C Sequencing

High‐quality genomic DNA extracted from muscle tissue of the reference individual was used to construct Hi‐C libraries. The DNA was digested with 100 U DpnII and incubated with Biotin‐14‐dATP. Ligated DNA was sheared into ∼400 bp fragments, followed by blunt‐end repair, A‐tailing, and purification via biotin–streptavidin pull‐down. The Hi‐C libraries were then quantified and sequenced on the DNBSEQ‐T7 platform [74].

This library generated a total of ∼217.12 Gb of raw sequencing data, comprising 1.447 × 10^9^ read pairs. After quality filtering, 200.91 Gb of clean data (1.436 × 10^9^ read pairs) were retained, with Q30 and Q20 scores of 97.9% and 93.5%, respectively, and a GC content of 41.77%.

### 5.7 CUT&Tag

Nine fresh muscle samples were collected from nine adult male *R. sinicus* individuals from Jiangxi Province, China (115.76°E, 28.80°N), comprising three biological replicates for each of three histone modifications (H3K27ac, H3K27me3, and H3K4me3). CUT&Tag libraries were prepared following a standard protocol with minor modifications. Briefly, samples were mildly fixed, conjugated to ConA‐coated magnetic beads, and permeabilized with 0.1% Triton X‐100. Primary antibodies were incubated at 16°C18°C for 2 h, followed by incubation with rabbit anti‐mouse IgG secondary antibodies at room temperature for 1 h. Tn5‐mediated tagmentation was then performed. DNA fragments were purified and amplified using 14 PCR cycles with NEBNext High‐Fidelity 2× PCR Master Mix, together with universal i5 primers and barcoded i7 primers. Libraries were purified using Agencourt AMPure XP beads to remove fragments shorter than 100 bp and sequenced on an Illumina NextSeq 500 platform with 75‐bp paired‐end reads.

Raw sequencing data were quality‐controlled using fastp (v0.23.4) and aligned to the *R. sinicus* T2T‐RSC1.0 genome using Bowtie2 (v2.3.5.1) [76] (–phred33 ‐I 10 ‐X 700). PCR duplicates were marked using sambamba (v0.8.2) [77] markdup, and duplicate as well as low‐quality reads were removed using SAMtools (v1.21) view. IgG‐CUT&Tag libraries were used as negative controls; high‐confidence peaks for each histone modification were identified using MACS2 (v2.2.7.1) [78] with a *q*‐value cutoff of 0.05. Genome‐wide signal tracks were generated from deduplicated BAM files and converted to BigWig format. Visualization of histone modification landscapes along the Y chromosome and chromosome 2 was performed using pyGenomeTracks (v3.9) [79].

### 5.8 ATAC‐Seq

Fresh liver and muscle tissue from *R. sinicus* were processed for ATAC‐seq. Cells were lysed using RSB buffer supplemented with detergent, and nuclei were collected by centrifugation. Transposition reactions were performed using Tn5 transposase, followed by DNA purification, PCR amplification, and size selection with AMPure XP beads. Libraries were sequenced on an Illumina platform.

Sequencing reads were quality‐filtered using fastp (v0.23.4), yielding high‐quality data with Q20 and Q30 scores of 97.12% and 91.36%, respectively, and an average sequencing depth of 115.84 million reads per library. Clean reads were aligned to the *R. sinicus* T2T‐RSC1.0 reference genome using Bowtie2 (v2.3.5.1). PCR duplicates were marked using sambamba (v0.8.2) markdup and removed together with low‐quality reads using SAMtools (v1.21) view (‐F 1804 ‐f 2 ‐q 30). Based on deduplicated alignments, chromatin accessibility signals were computed using BEDTools (v2.31.1) [80] genomecov and converted to BigWig format. Accessibility landscapes along the Y chromosome and chromosome 2 were visualized using pyGenomeTracks (v3.9) to characterize regional chromatin openness and its relationship with histone modification patterns.

### 5.9 BioNano

High‐molecular‐weight genomic DNA was extracted from bat muscle tissue using the BioNano Prep Animal Tissue DNA Isolation Kit. DNA molecules were labeled using the Direct Label Enzyme (DLE‐1) from the Bionano Prep DLS Labeling DNA Kit and processed according to the Bionano Saphyr workflow. Labeled DNA molecules with a minimum length of 150 kb and at least 9 labels per molecule were loaded onto the Bionano Saphyr system and imaged at GrandOmics.

The raw dataset contained 11,136,301 DNA molecules, of which 4,921,070 passed quality filtering. The total length of filtered molecules was ∼1.21 Tb, with an average molecule length of 246.2 kb and an N50 of 248.6 kb. For molecules ≥150 kb with ≥9 labels, the total DNA length was ∼1.19 Tb, and the N50 was 249.4 kb. The average label density was 15.3 labels per 100 kb, with an average backbone signal‐to‐noise ratio of 27.97. Mapping to the reference genome yielded an alignment rate of 85.2% and an effective coverage of 472.9×.

### 5.10 Iso‐Seq

Total RNA was extracted from eight tissues (wing membrane, heart, muscle, stomach, kidney, spleen, liver, and lung) collected from six *R. sinicus* individuals (4 males and 2 females) and used for PacBio Iso‐Seq full‐length transcriptome sequencing. A total of 7,004,044 polymerase reads were generated, comprising 484.7 Gb of sequence data, with a mean read length of 69,205 bp and an N50 of 141,382 bp. Raw subreads were processed using SMRT Link (v3.1.1; https://github.com/WenchaoLin/SMRT‐Link) to generate circular consensus sequencing (CCS) reads. All CCS reads met the Q20 quality threshold, and 78.09% met the Q30 threshold. CCS reads were further processed using IsoSeq3 (v4.2.0, https://github.com/PacificBiosciences/IsoSeq), including primer removal and barcode trimming, followed by clustering with parameters set to a minimum transcript length of 500 bp to generate non‐redundant full‐length non‐chimeric (FLNC) transcripts.

FLNC transcripts were aligned to the reference genome using GMAP (v2021‐12‐17) [81], w retaining alignments with a mapping quality score ≥ 20. Iso‐Seq transcript evidence was integrated with existing gene models using EVidenceModeler (v2.1.0) [82] to refine exon boundaries, supplement alternative splicing isoforms, and validate gene structures. Manual curation was performed using the Integrative Genomics Viewer (IGV, v2.10.3) [83], with a particular focus on the Y chromosome. Genes with transcript structures inconsistent with Iso‐Seq evidence were corrected or removed, and novel genes supported by Iso‐Seq data were added. In total, 131 protein‐coding genes and five pseudogenes were annotated on the Y chromosome.

### 5.11 RNA‐Seq

RNA was extracted from the wing membrane, heart, muscle, stomach, kidney, spleen, liver, and lung tissues of *R. sinicus* individuals. Additional testicular RNA samples were obtained from four male individuals, with RNA integrity numbers ranging from 5.6 to 8.8. Complementary DNA libraries were prepared using the TruSeq RNA Library Preparation Kit and sequenced on an Illumina platform. Raw sequencing reads were quality‐filtered using fastp (v0.23.4). Clean reads were aligned to the soft‐masked version of the *R. sinicus* T2T‐RSC1.0 reference genome using HISAT2 (v2.2.1) [84]. Transcript assembly and expression quantification were performed using StringTie (v2.2.3) [85] with the parameters ‐e and ‐A. Differential expression analysis was performed using DESeq2 (v1.44.0) based on a negative binomial generalized linear model. Read counts were normalized using the median‐of‐ratios method implemented in DESeq2. Biological replicates were included in the statistical model to estimate biological variation. Multiple testing correction was performed using the Benjamini–Hochberg false discovery rate (FDR) method. Genes with an absolute log_2_ fold change (|log_2_FC|) > 1 and a Benjamini–Hochberg‐adjusted *P*‐value < 0.05 were considered differentially expressed genes (DEGs).

### 5.12 Single‐Cell Sequencing

Two adult male *R. sinicus* were euthanized for single‐cell suspensions. Each individual was processed independently as a biological replicate, and samples from different individuals were not pooled during cell preparation or library construction. Individual identities were retained throughout downstream single‐cell analyses. Libraries were constructed by using the single‐cell instrument (10x Genomics, Pleasanton, CA) with the Chromium v2 single‐cell 3’ library and gel bead kit V2 (10x Genomics, Pleasanton, CA). In brief, cell suspensions were diluted in DPBS with 0.04% BSA to a concentration of 1000 cells/mL, and the concentration was measured with a hemocytometer. The volume of single‐cell suspension required to generate 4000 single‐cell gel beads in emulsion (GEMs) was loaded into a separate channel on the single‐cell 3′Chip. The final libraries were qualified with an Agilent 2100 Bioanalyzer (Agilent Technologies, Santa Clara, CA, USA) and quantified by qPCR with the quantification kit (Tian Gen company, China) for Illumina with the QuantStudio 12K Flex Real‐time PCR system (Thermo Fisher Scientific, Waltham, MA, USA). Libraries were diluted to 2 nm in each and pooled with equal volumes before being sequenced on HiSeq X Ten (Illumina, Inc., San Diego, CA, USA) with 150‐bp paired‐end strategies.

### 5.13 Autosome Assembly

A preliminary genome assembly was generated from HiFi reads using hifiasm (v0.24.0‐r702) [86, 87, 88] with default parameters. Assemblies integrating both HiFi and ONT reads were also performed with hifiasm (v0.24.0‐r702), while ONT‐only assemblies were generated using NextDenovo (v2.5.2) [89]. The completeness of the three preliminary assemblies was assessed with BUSCO (v5.1.3) [90] based on the mammalia_odb10 database (2024). To assess assembly quality, 21‐mer databases were constructed from short reads and HiFi reads using meryl (v1.4.1) [91], and genome quality values (QV) were calculated using Merqury (v1.3) [92]. Assemblies were further aligned to the reference genome with minimap2 (v2.28‐r1209) [92, 93] and visualized via synteny dotplots using paf2dotplot (https://github.com/moold/paf2dotplot). Assemblies were compared based on contiguity, total length, number of contigs, sequence consistency relative to the reference, and BUSCO (v5.1.3) score. Across metrics, the hybrid assembly consistently outperformed the HiFi‐only and ONT‐only assemblies and was therefore selected for downstream analyses.

FCS‐GX (v0.5.5) [94] was employed to detect and remove contamination from the assembled genome. The screen genome module of FCS‐GX (v0.5.5) was first applied to identify contaminant sequences in the preliminary assembly using the reference database (gxdb) and the NCBI taxonomy ID of the target species (89,399), generating a contamination report. Then, the clean genome module was used to remove potential contaminant sequences according to the detection report, producing a cleaned genome (clean FASTA) and contaminant sequence file (contam FASTA), providing a contamination‐free reference genome for subsequent analysis.

BWA (v0.7.18) [95, 96, 97] was used to index the decontaminated genome, and paired‐end Hi‐C sequencing data were aligned to the decontaminated genome, generating the alignment file (Hi‐C BAM). During alignment, PCR duplicates were removed using Samblaster (v0.1.26) [98], and SAMtools (v1.21) was used to filter high‐quality alignments. Subsequently, HapHiC (v1.0.5) [99, 100, 101] was used to further filter the Hi‐C alignments (NM ≤ 3), producing high‐quality Hi‐C data files. Chromosome‐level scaffolding and optimization were performed using the HapHiC (v1.0.5) pipeline, yielding a genome with 18 gaps. The scaffolded genome with Hi‐C data was then visualized and manually refined using Juicebox (https://github.com/aidenlab/Juicebox), producing chromosome‐level assembly sequences. Finally, reference‐guided alignment corrections and validation were performed using minimap2 (v2.28‐r1209), allowing identification and classification of autosomes and outputting the final autosomal sequences.

### 5.14 X/Y Chromosome Assembly

During the assembly of sex chromosomes, haplotype‐resolved contigs were first generated using hifiasm (v0.24.0‐r702) based on Nanopore and PacBio HiFi long‐read sequencing data in combination with Hi‐C data. These contigs were then anchored and ordered with the aid of Hi‐C data to construct a chromosome‐level genome using HapHiC (v1.0.5) and manually curated with Juicebox (https://github.com/aidenlab/Juicebox), which was further refined using the reference genome. Finally, minimap2 (v2.28‐r1209) was employed to align the results, enabling accurate identification of the sex chromosomes and yielding complete sex chromosome sequences.

### 5.15 Chromosome Validation

The final autosome sequences and sex chromosome sequences were merged. Meanwhile, HiFi reads and Nanopore ultra‐long reads were mapped to the preliminary chromosome‐level genome using minimap2 (v2.28‐r1209) with the parameters ‐x map‐hifi and ‐x map‐ont, respectively. We identified positions that deviated from the reference genome. Each discrepancy was inspected to distinguish assembly errors from true biological variation. Errors were corrected through scaffolding revisions, while validated positions were retained based on inspection in IGV (v2.10.3), producing the final chromosome‐level genome. Subsequent gap filling, polishing, and telomere assembly were performed to generate the complete raw‐T2T genome.

### 5.16 Gap Filling and Telomere Assembly

Based on v4 assembly, gaps were filled using HiFi and ONT ultra‐long reads (≥50 kb) to generate the gap‐free genome. The resulting assembly was validated through read mapping (Nanopore ultra‐long reads, HiFi reads, MGI paired‐end reads) and synteny analysis against the reference genome. Assemblies passing validation were retained as the gap‐free genome. The detailed workflow is summarized in the gap closure flowchart (see detailed gap closure workflow in Figure S5) and as follows.

Ultra‐long and HiFi reads were mapped to the genome using minimap2 (v2.28‐r1209) with parameters (‐ax map‐ont –secondary = no for ONT reads and ‐ax map‐hifi –secondary = no for HiFi reads). Reads flanking each gap were selected as anchor reads, which had to be uniquely mapped with mapping identity ≥ 99% and mapping coverage ≥ 99%, and be the first reads adjacent to the gap on both sides (see “red reads” in the workflow diagram). Unmapped reads (excluding those containing telomeric features, that is, reads with more than 20 consecutive telomeric repeats) and reads mapping within the coordinate range defined by the paired anchor reads flanking the gap were collected and mutually aligned to identify overlaps between reads. Extension paths were built based on the overlap relationships with anchor reads and among other reads until the reads from both sides of the gap overlapped. The extended reads were assembled using the NextGraph module of NextDenovo (v2.5.2) based on their overlap relationships, and the resulting contig was used to replace the corresponding gap region. The assembled genome after telomere completion was subjected to base error correction using Nextpolish2 (v0.1.0) [102]. Reads were remapped to this corrected genome using minimap2 (v2.28‐r1209) to identify regions with significantly abnormal coverage (regions with fewer than 5 “perfectly mapped” reads, where “perfectly mapped” reads are defined as uniquely mapped with identity > 90% and coverage > 90%). If no issues were found, the genome was designated as the pre‐T2T assembly.

Sequencing ultra‐long (≥50 kb) and HiFi reads were mapped to the gap‐filled genome using minimap2 (v2.28‐r1209) with parameters (‐x map‐ont –secondary = no for ONT reads and ‐x map‐hifi –secondary = no for HiFi reads). Reads mapping to the chromosome termini were selected as the anchor reads, which must be uniquely mapped with mapping identity ≥99% and mapping coverage ≥99%, and be the innermost reads adjacent to the unassembled telomere. Additionally, reads containing telomeric repeat units were also used as anchor reads. Unmapped reads and anchor reads were mutually aligned to identify overlaps between reads. Starting from the anchor reads mapping to the chromosome termini, extension paths were built using the NextGraph module of NextDenovo (v2.5.2) based on the overlap relationships with anchor reads and among other reads, until reads containing telomeric repeat units were reached. The assembled sequence from the extended path was used to fill the missing telomeric sequence at the chromosome terminus to form a gap‐free genome, and then Nextpolish2 (v0.1.0) was used to polish the genome to produce a pre‐T2T genome.

### 5.17 Centromere Region Identification

Multiple genomic features were analyzed to identify candidate centromere regions, including repeat sequence density (>85%) calculated in 100 kb windows based on repeats identified by RepeatMasker (v4.1.7) [103], satellite DNA density (>1%) in 100 kb windows, and sequences with high transposable element (TE) content identified by RepeatMasker (v4.1.7). KMC (v3.2.4) [104, 105, 106] was used to count k‐mers, and SRF (https://github.com/lh3/srf) [107] was applied to select repeat sequences of specific length (≥20 bp). These sequences were then aligned to the reference genome using BLASTN (v2.16.0) [108] to obtain genomic locations, followed by merging and filtering. For each chromosome, the longest candidate region (identity > 90%) was retained as a candidate centromeric region. Meanwhile, the distribution of SRF elements was quantified in fixed windows to calculate coverage density (100 kb windows, >0). BED intervals corresponding to these features were separately extracted. All feature intervals were merged, and candidate region lengths for each chromosome were calculated. These candidate regions were compared with Topologically Associating Domain (TAD) maps to obtain predicted centromere regions for each chromosome, and a final file containing the candidate centromeric regions was generated.

### 5.18 rDNA Identification

RNAmmer (v1.2) [109] was used to predict rDNA in the T2T‐RSC1.0 genome, generating annotations in GFF format. The chromosome, start, and end positions, and length information of rDNA loci were then extracted using awk.

### 5.19 Segmental Duplication (SD) Identification

The assembly with repeats masked by RepeatMasker (v4.1.7) was used for SD detection by BISER (v1.4) [110] with parameters “–max‐error 20 –max‐edit‐error 10 –gc‐heap 3G –kmer‐size 21”. Then, SDs were filtered based on the following criteria: mismatch rate ≤ 10, gap rate ≤ 50, and the length of the first SD segment divided by the length of the second segment >90%. Subsequently, SD regions were classified based on their lengths.

### 5.20 Methylation by PacBio Long Reads

Cytosine methylation was detected based on raw HiFi data. Pbmm2 (v1.16.99; https://github.com/PacificBiosciences/pbmm2) was used to align BAM‐format HiFi data to the T2T‐RSC1.0 assembly. Subsequently, the “aligned_bam_to_cpg_scores” command in pb‐CpG‐tools (v2.3.2; https://github.com/PacificBiosciences/pb‐CpG‐tools) was used to generate site‐specific 5mC methylation probabilities. Methylation sites with frequency >50% were selected, and BEDTools (v2.31.1) was used to calculate methylated base frequencies within 100 kb windows. The distribution was visualized using the R package karyoploteR (v1.8.4) [111].

### 5.21 Genome Assembly Validation and Quality Assessment

T2T‐RSC1.0 assembly was compared with the bat reference genome RSC01 (GCA_036562045.1). Synteny dotplot analysis was performed after alignment using minimap2 (v2.28‐r1209) with the parameter “‐cx asm10”. The synteny dotplots between the two assemblies were visualized by paf2dotplot (https://github.com/moold/paf2dotplot).

Depth coverage was calculated by PanDepth (v2.25) [112] in 100 kb windows, based on BAM files generated by minimap2 (v2.28‐r1209) with parameter “‐ax map‐hifi” for HiFi and ONT long reads, and with parameter “‐ax sr” for short reads. Genome coverage was visualized using the karyoploteR package (v1.8.4).

Using Merqury (v1.3) with the meryl (v1.4.1) command, 21‐mer databases were created separately from short reads and HiFi long reads. Based on these, quality value (QV) scores and erroneous k‐mer frequencies for T2T‐RSC1.0 were calculated.

Assembly quality was assessed using QUAST (v5.2.0) [113] both against the specified reference genome (GCA_036562045.1) and in a reference‐free mode.

Assembly completeness of T2T‐RSC1.0 was assessed with BUSCO (v5.1.3) using the mammalia_odb10 (2024) and laurasiatheria_odb10 (2024) lineage datasets.

The minUniqueKmer (v1.0) [114] software was used to calculate the MUK length of the genome. The MUK length reflects the minimum distance required to uniquely identify a sequence within the genome. In the visualization, blue regions indicate sequences unchanged from the previous version, purple indicates sequences with partial similarity, red marks gap regions, and yellow and green highlight completely novel sequences.

### 5.22 Previously Unresolved Region (PUR) Identification

Winnowmap (v2.03) [115, 116] was used to align the T2T genome to the reference genome. Subsequently, BEDTools (v2.31.1) was applied to sort, merge, and perform complement operations on the alignment results, ultimately obtaining PURs on the reference genome that were not covered.

### 5.23 Repeat Annotation

GMATA (v2.3) [117] and Tandem Repeats Finder (TRF; v4.09) [118] were used to perform de novo prediction of tandem repeat sequences. The de novo repeat library was merged with the Repbase repeat sequence database. Then, RepeatMasker (v4.1.7) was used with default parameters to annotate repeats based on the merged repeat library. RepeatMasker (v4.1.7) was applied to mask repeats in the T2T‐RSC1.0 assembly, and the masked assembly was used for subsequent SD analysis.

### 5.24 Annotation of Protein‐Coding Genes

For the assembled T2T‐RSC1.0 genome, we integrated three methods, transcriptome‐based prediction, de novo prediction, and homology‐based prediction–to annotate protein‐coding genes. The specific workflow was as follows: (1) Transcriptome‐based prediction: First, raw transcriptome data were subjected to quality control and filtering using fastp (v0.23.4). Then, HISAT2 (v2.2.1) and StringTie (v2.2.3) were used for transcript assembly, followed by gene prediction using TransDecoder (v5.7.1) [119]. (2) De novo prediction: Based on the mapping results from transcriptome‐based prediction, the PASA (Program to Assemble Spliced Alignment) pipeline (v2.5.3) [120] was used to generate a high‐quality training dataset. This dataset was then applied to train parameters for prediction software such as Augustus (v3.5.0) [121] and GeneMark‐EPT (v1.02) [122]. (3) Homology‐based prediction: Genomes of multiple species were downloaded from the NCBI database as references, including model species (*Homo sapiens; H. sapiens*, *Canis lupus*, *Bos taurus; B. taurus*, and *Equus caballus; E. caballus*), four bat species with published genomes (*Myotis myotis; M. myotis*, *R. ferrumequinum*, *Cynopterus sphinx; C. sphinx*, and *Rhinolophus affinis; R. affinis*) (Table S28). Homology‐based gene annotation was performed using GeMoMa (v1.9) [123].

Finally, we integrated the gene prediction results from the above three methods using EvidenceModeler (v2.1.0) to obtain preliminary predictions. To further refine the annotations, we generated transcript and protein sequence files for each species using the same annotation files and genome sequences as in the homology‐based prediction. These were supplemented and manually revised via IGV (v2.10.3) using Eviann (v2.0.2) [124]. The completeness of the final annotation results was assessed using the mammalia_odb10 gene set from BUSCO (v5.1.3).

### 5.25 Gene Function Annotation

Predicted gene sequences of the T2T‐RSC1.0 were subjected to sequence similarity searches using BLASTP (v2.16.0) [125] against the SwissProt and the non‐redundant protein (nr) database. Additionally, InterProScan (v5.71‐102.0) [126] was employed for annotations such as Gene Ontology (GO), Pfam, and Conserved Domain Database (CDD), with the run parameters set as ‐goterms, ‐t p, and ‐pa.

### 5.26 Variant Calling

To evaluate short‐read alignment and variant calling using T2T‐RSC1.0 and RSC01 as reference genomes, respectively, whole‐genome sequencing data from 52 bat individuals were collected. fastp (v0.23.4) was used to remove low‐quality bases and reads. High‐quality paired‐end reads were aligned separately to T2T‐RSC1.0 and RSC01 using BWA mem (v0.7.18) with default parameters. Aligned reads were converted to BAM format, sorted using SAMtools (v1.21), and sequencing depth and alignment statistics were summarized using SAMtools (v1.21) “stats” module [19].

Single‐nucleotide polymorphisms (SNPs) for individuals were called from the BAM files using the HaplotypeCaller module in GATK (v4.3.0) [127, 128, 129, 130]. Joint genotyping was performed with the GenotypeGVCFs module in GATK (v4.3.0). To reduce potential false positive calls, SNPs were filtered using the VariantFiltration module in GATK (v4.3.0) with the following parameters: QUAL < 30 || QD < 2 || MQ < 40 || FS > 60 || SOR > 3 || MQRankSum < −12.5 || ReadPosRankSum < −8. Filtered results were further filtered by bcftools (v1.21) [131] to select high‐quality variant sites. SnpEff (v5.2e) [132] was used to annotate VCF files generated using T2T‐RSC1.0 and RSC01 as reference genomes, respectively [19].

Structural variants (SVs) were called from BAM files using three tools: Delly (v1.3.3) [133], Manta (v1.6.0) [134], and Smoove (v0.2.8; https://github.com/brentp/smoove), all with default parameters. SV sizes between 50 bp and 1 Mb and supported by at least two tools were selected. All individuals’ SVs were merged using SURVIVOR (v1.0.6) [135] with a merging window of 1 kb and minimum SV length of 50 bp [19]. Based on these results, the number of INDELs in the satellite regions, PUR regions, and non‐PUR regions of the T2T‐RSC1.0 and RSC01 genomes were counted separately.

### 5.27 Population Genetic Structure

High‐quality SNPs were used for population genetic structure analysis. For the whole‐genome SNP dataset, PLINK (v1.9) [136] was used with the parameter “‐indep‐pairwise 50 5 0.5” to remove linked SNP sites. For Y chromosome data, SNPs were first extracted from the whole‐genome SNP dataset using bcftools (v1.21) without linkage pruning. Principal component analysis (PCA) was performed using PLINK (v1.9) on both the whole‐genome SNP dataset and the Y chromosome SNP dataset. The linkage‐pruned SNP data from PLINK (v1.9) were used as input for ADMIXTURE (v1.3.0) [137] to analyze population structure across different numbers of clusters (*K* = 1–7). Cross‐validation (–cv) was employed to calculate CV errors for each *K‐*value, and the optimal *K* was determined based on the minimum CV error. Results were visualized using R. Phylogenetic relationships based on the obtained variant sites were investigated. The best‐fit model identified by ModelFinder using Bayesian Information Criterion (BIC) was TVM+F+R2. Maximum likelihood tree construction was performed using IQ‐TREE (v2.4.0) [138, 139, 140] with 1000 bootstrap replicates and visualized by iTOL (v6.7.1; https://itol.embl.de/).

### 5.28 Population Demographic History

The target species’ genome was first split into single chromosome sequences using LASTZ (v1.04.22) [141], and whole‐genome alignments were performed against the genome of *R. affinis*, a closely related species of *R. sinicus*. Based on the alignment results, the total number of bases and the number of mutation sites were calculated, from which genetic distance (D) and mutation rate, derived from generation time and divergence time, were estimated. A generation time (*g*) of 7.49 years was used based on Pacifici et al. [142], and a mutation rate (*μ*) of 1.7 × 10^−^^8^ substitutions per site per generation was used for the PSMC analysis. The mutation rate was estimated from the nucleotide divergence between *R. sinicus* and *R. affinis* based on syntenic genomic regions, using a divergence time of 35 Ma obtained from TimeTree. Next, to reasonably set depth filtering parameters in PSMC analysis, the average sequencing depth was calculated from BAM files of resequencing data using SAMtools (v1.21). Typically, the minimum depth (‐d) and maximum depth (‐D) were set to one‐third and twice the average sequencing depth, respectively, to ensure data quality. High‐quality consensus sequences (consensus FASTQ) were generated from BAM files using bcftools (v1.21) and converted to PSMC input format using the fq2psmcfa script provided by PSMC (v0.6.5‐r67) [143]. The main PSMC model was then run to infer population demographic history. One hundred bootstraps were performed based on segmented sequences to assess the stability and confidence of the inference results. Finally, combining the estimated mutation rate (m) and generation time (g), population history curves including confidence intervals were plotted using thepsmc_plot.pl script. This revealed the changes in effective population size of the target species over millions of years, providing a scientific basis for evolutionary and conservation studies.

### 5.29 Major Histocompatibility Complex (MHC) Refinement

We retrieved the positions of the MHC regions in humans, rats, mice, bats via the UCSC Genome Browser (https://genome.ucsc.edu/), and downloaded the immune gene sequences within these regions by species, including HLA genes, RT1 genes, and H2 genes. Using minimap2 (v2.28‐r1209), we aligned the T2T‐RSC1.0 genome with the previously downloaded MHC segments from well‐studied model species. Intervals with a similarity higher than 90% and continuity were selected as candidate MHC regions. Chromosome sequences containing these candidate regions were extracted and further aligned to the corresponding loci from other species to confirm the genomic positions and order of MHC class I and II genes in T2T‐RSC1.0. To validate MHC gene content and structure, multi‐tissue full‐length transcriptome data were integrated. Candidate gene loci were manually inspected and corrected to ensure accurate annotation of MHC class I and class II genes, confirming the authenticity of the MHC segments and loci in *R. sinicus*.

### 5.30 Viral Genome Discovery, Abundance Quantification, and Diversity Analysis

Viral metagenomic sequencing was performed on intestinal tissues from 52 *R. sinicus* individuals. For each sequencing library, raw reads were quality‐filtered using Trimmomatic (v0.39) [144] with parameters (LEADING:5 TRAILING:5 SLIDINGWINDOW:4:15 MINLEN:36). Cleaned reads were de novo assembled into contigs using MEGAHIT (v1.1.3) [145] with default parameters. Viral contigs were identified by comparing all assembled contigs against the nr database using DIAMOND BLASTx (v0.9.24.125; *e*‐value ≤ 1 × 10^−^^5^) [108]. Contigs taxonomically classified within the “Viruses” kingdom based on top BLAST hits were initially designated as potential viral sequences. To filter out host genome sequences, endogenous viral elements, and artificial vectors, these potential viral contigs were subsequently aligned against the non‐redundant nucleotide (nt) database using BLASTN (v2.16.0).

Quality‐trimmed reads were sequentially aligned against the SILVA database (v132.1) and the T2T‐RSC1.0 using Bowtie2 (v2.3.5.1) to remove ribosomal RNA‐associated and host‐derived reads. The remaining reads from each library were then mapped to the assembled contigs using Bowtie2 (v2.3.5.1) in end‐to‐end alignment mode with sensitive parameters. Read counts for each contig were obtained using SAMtools (v1.21) for sorting and indexing the alignments. Viral RNA read counts were normalized within libraries as reads per million total non‐rRNA reads (RPM) to enable cross‐geographical library comparisons, applying an abundance threshold of RPM > 1 to minimize false positives. Subsequently, the Shannon diversity index (α‐diversity) was calculated and compared across geographic regions using the vegan package, with statistical significance of differences in Shannon indices assessed by Hutcheson's *t*‐test.

### 5.31 Synteny Visualization

Each chromosome was first aligned to the reference genome using minimap2 (v2.28‐r1209). SyRI (v1.7.0) [146] was then applied to determine the forward and reverse strand orientation of each chromosome to ensure accurate alignment. Chromosome sequences with adjusted orientations were merged, followed by a whole‐genome minimap2 (v2.28‐r1209) alignment and SyRI analysis. Plotsr (v1.1.1) [147] was finally used to visualize structural variations, enabling comprehensive detection and display of genome‐wide structural variants.

### 5.32 Hi‐C 3D Heatmap and TAD

Cleaned Hi‐C sequencing data were aligned to the T2T‐RSC1.0 genome using BWA (v0.7.18), and sorted BAM files were generated using SAMtools (v1.21). Restriction sites were generated using hicFindRestSite (https://github.com/deeptools/HiCExplorer) [148, 149] based on enzyme cutting sites (restriction enzyme site file). Hi‐C contact matrices based on restriction enzyme sites were constructed using hicBuildMatrix (https://github.com/deeptools/HiCExplorer) with a bin size of 100 kb. The contact matrices were normalized and corrected for biases and outliers using hicCorrectMatrix (https://github.com/deeptools/HiCExplorer), resulting in corrected Hi‐C matrices. Hi‐C heatmaps at the whole‐genome and single‐chromosome levels were plotted using hicPlotMatrix (https://github.com/deeptools/HiCExplorer) to display spatial proximity relationships across chromosomal regions. TADs were identified using hicFindTADs based on Hi‐C contact matrices at a resolution of 40 kb (https://github.com/deeptools/HiCExplorer). TAD visualization plots integrating Hi‐C data and other omics tracks were generated by hicPlotTADs, facilitating understanding of functional chromosomal 3D structural domains.

### 5.33 Identification of Y Chromosome Functional Units

Syntenic regions between sex chromosomes were identified using NGenomeSyn (v1.41) [150]. Minimap2 (v2.28‐r1209) was jointly used to align the X and Y chromosomes, and regions with sequence similarity >99% were annotated as PARs [19]. Candidate heterochromatic regions were defined as overlapping regions of SRF (Scaffold Recognition Feature), satellite sequences, and TADs. Referring to the study by Skaletsky et al. [33], heterochromatic regions were required to contain >80% satellite repeat sequences. CUT&Tag‐seq and ATAC‐seq data were further integrated to confirm non‐open chromatin blocks on the Y chromosome, assisting in the final delineation of heterochromatic regions [29, 33].

The MSY is a core segment for sexual differentiation, encompassing key functional regions such as ampliconic regions and X‐degenerate regions [33]. The Y chromosome of *R. sinicus* was meticulously delineated following the annotation standards defined by Skaletsky et al. [33]. Based on repeat sequence annotation results, non‐PAR segments of the T2T‐RSC1.0‐Y assembly were hard‐masked (sequences annotated as satellites, LINEs, LTR elements, and DNA elements were masked) and divided into 5 kb sliding windows (2 kb step size). These window sequences were used as queries to map back to the MSY segment via BLAST (v2.16.0) [108] (parameter: ‐perc_identity 50). After removing self‐aligned regions, adjacent regions <100 kb were merged into continuous fragments, and false‐positive fragments <90 kb were excluded. The remaining regions were annotated as ampliconic regions [32, 33]. A triangular heatmap of Y chromosome self‐alignment similarity was constructed using the StainedGlass (v0.6) [151] with a 10 kb window size. Regions were retained as ampliconic candidates only when they satisfied both thresholds of sequence similarity > 85%; and overlap with the previously defined candidate regions. Finally, the regions that met these criteria were annotated as ampliconic regions [71].

After excluding PARs, ampliconic regions, and heterochromatic regions, the remaining Y chromosome sequences were initially marked as potential X‐degenerate regions. OrthoFinder (v2.5.5) [152] was used to analyze orthologous genes on the Y chromosomes of humans, gorillas, chimpanzees, sheep, and *R. sinicus*, determining the distribution of single‐copy homologous protein sequences on the *R. sinicus* Y chromosome. By determining the precise distribution of orthologous single‐copy protein sequences on the Y chromosome of the Chinese rufous horseshoe bat, comparing their distribution within regions of closely related species, and integrating these results with high‐similarity overlapping blocks identified in a triangular similarity heatmap, we ultimately refined and improved the boundary delimitation of the X‐degenerate regions [32].

Based on the above alignment and annotation analyses, species‐specific X‐transposed regions (XTRs) were identified. Hard‐masked MSY sequences of the Y chromosome were aligned with 5 kb sliding windows of the X chromosome via BLAST (v2.16.0); regions with sequence identity ≥94% and length > 1 kb were annotated as XTRs [32]. To verify the presence of human‐like XTRs in bats, LASTZ (v1.04.22) was used to align the XTR segment of the human Y chromosome (referenced to T2T‐CHM13 v2.0) with the bat T2T‐RSC1.0‐Y assembly, and high‐similarity regions were identified as XTRs [32].

Relying on the existing self‐alignment heatmap of the Y chromosome and the results of Y chromosome partitioning, first, the overall distribution characteristics of repetitive sequences on the Y chromosome were counted. Subsequently, two types of key segments were focused on and screened as candidate regions for composite elements: one was regions with high density of repetitive sequences, and the other was regions where the similarity between repetitive sequences within the region was relatively low and which contained repeat units composed of three or more repetitive sequences [29]. Furthermore, RNA‐seq and ISO‐seq transcriptome data were combined to conduct functional verification on the candidate regions. Finally, regions with no detectable gene expression signals were regarded as composite elements.

### 5.34 Evolutionary Strata and X–Y Gametolog Analysis

To obtain alignment outputs of homologous regions between X and Y chromosomes, X and Y sequences were first hard‐masked. LASTZ (v1.04.22) was used for pairwise alignment (parameters: –step = 19 –hspthresh = 2200 –inner = 2000 –ydrop = 3400 –gappedthresh = 10000 –format = axt). Low‐quality alignment fragments were filtered and optimized using chainPreNet (UCSC v469) [153]; chainNet (UCSC v469) and netSyntenic (UCSC v469) were used to construct an alignment network; finally, netToAxt (UCSC v469) was used to extract syntenic alignment regions of sex chromosomes [154]. Based on high‐quality syntenic regions, a 100 kb sliding window was used to align along the Y chromosome, capturing sequence divergence signals and similarity between sex chromosomes to assist in defining the boundaries of different evolutionary strata, providing data support for analyzing the evolutionary history of sex chromosome differentiation [155, 156].

BLAST (v2.16.0) was used for bidirectional alignment to initially screen for sex chromosome homologous genes (parameters: ‐evalue 1e‐5, ‐max_target_seqs 1). MAFFT (v7.525) [157] was used for the alignment analysis of gametologous protein sequences. PAL2NAL (v14.1) [158] was employed to back‐translate the protein alignment results into codon‐aligned gametologous CDS sequences. Before calculation, the gene pair list was prepared, and the corresponding CDS/protein sequences were extracted from the genome to ensure the analysis objects were accurate. Then, KaKs_Calculator (v3.0) [159] was used to calculate the synonymous substitution rate (*dS*) for each gene pair (parameter: ‐m MA). Finally, based on the pairwise dS values and their positional distribution in humans, gametologous gene pairs were grouped into different evolutionary strata [34, 57, 159].

### 5.35 CLDN34‐Focused Analysis

Within this general framework, the *CLDN34* gene family was subjected to further in‐depth analysis due to its pronounced copy number expansion on the Y chromosome. All annotated *CLDN34* coding sequences were extracted and translated into protein sequences. Multiple sequence alignment of *CLDN34* proteins was conducted using MAFFT (v7.525), followed by codon‐aware back‐translation with PAL2NAL (v14.1), ensuring preservation of reading‐frame integrity.

The resulting codon‐aligned CDS sequences were used to reconstruct a maximum‐likelihood phylogeny in IQ‐TREE (v2.4.0), with automatic model selection and node support assessed using 1000 ultrafast bootstrap replicates and 1000 SH‐aLRT tests. The resulting phylogenetic tree was visualized with iTOL (v6.7.1; https://itol.embl.de/) to enhance the clarity of topological structures, with branches color‐coded to distinguish X‐linked and Y‐linked *CLDN34* copies, and bootstrap/SH‐aLRT support values annotated at key nodes. This phylogenetic analysis enabled the resolution of evolutionary relationships among CLDN34 paralogs, allowing us to infer duplication history, lineage‐specific expansions, and the evolutionary trajectory of Y‐linked CLDN34 copies in the context of sex chromosome differentiation.

### 5.36 Phylogenetic Analysis and Divergence Time Estimation

In addition to the T2T‐RSC1.0 genome, we also included nine bat species (*Pipistrellus kuhlii*, *Phyllostomus discolor*, *M. myotis*, *Molossus molossus*, *Rousettus aegyptiacus*, *R. ferrumequinum*, *C. sphinx*, *R. affinis*, *Rhinolophus pearsoni*) and five model species (*E. caballus*, *B. taurus*, *H. sapiens*, *Macaca mulatta*, *Mus musculus*) downloaded from the NCBI database and NGDC (Table S28). To ensure the stability and accuracy of subsequent analyses, only the longest transcript per gene was retained for downstream analyses. Single‐copy one‐to‐one orthologous gene families were identified among the 15 species using OrthoFinder (v2.5.5) based on the protein‐coding sequences.

Nucleotide sequences of the identified orthologs were aligned at the codon level using MACSE (v2.07) [160]. Alignment regions containing gaps or non‐homologous fragments were removed using trimAl (v1.5) [161]. The filtered alignments were concatenated into a supergene matrix for phylogenetic inference. A maximum likelihood (ML) phylogenetic tree was constructed using RAxML (v8.2.12) [162] with 1000 bootstrap replicates. Divergence times were estimated using MCMCtree (v4.10.7) [163, 164, 165]. We incorporated five fossil calibration points to constrain the molecular clock: the split between Hominoidea and Cercopithecoidea (23 to 33.9 Ma) [166], the divergence between Euarchontoglires and Laurasiatheria (95.3 to 113 Ma) [167], the divergence within Pteropodidae (20 to 29 Ma) [168], the divergence between Archonta and Glires (61.5 to 100.5 Ma) [166], and the origin of Chiroptera (58 to 71 Ma) [167].

### 5.37 Gene Family Expansion and Contraction

Gene family expansion or contraction analyses were performed by CAFE (v4.2.1) [169] based on the results from OrthoFinder (v2.5.5). Gene families showing significant expansion or contraction were identified using a Monte Carlo resampling procedure, with a significance threshold of *P* < 0.05. The results were summarized and visualized using the chiplot website (https://www.chiplot.online/tvbot.html).

### 5.38 Positive Selection Analysis

Rates of nonsynonymous (*dN*) and synonymous (*dS*) substitutions and their ratio (*ω* = *dN*/*dS*) were estimated for each orthologous gene using the CODEML program implemented in PAML (v4.10.7) [170], based on the species phylogeny inferred in this study. First, the branch‐site model was used for detecting positive selection genes (PSGs). Specifically, the *R. sinicus* and *Rhinolophus* lineage were specified as foreground branches. A likelihood ratio test (LRT) was used to compare the alternative branch‐site model, which allows a subset of sites to evolve under positive selection (*ω* > 1) along the foreground branch, against the null model that restricts *ω* to 1. Using the Bayes Empirical Bayes (BEB) analysis, positively selected sites (PSSs) were estimated with posterior probabilities ≥0.80. To minimize the potential effects of false positives, the median interval between PSSs is calculated, and alignments are removed if the median interval is <10 amino acids. Then, the branch model was used to identify REGs. The one‐ratio model assumed that all branches evolve at the same rate, while the two‐ratio model allowed the foreground branch to evolve at a higher rate than other branches. For both PSG and REG analyses, LRT‐derived *P* < 0.05 were adjusted for multiple testing using the Benjamini–Hochberg FDR procedure, and genes with corrected *q*‐values < 0.05 were retained as significant PSGs or REGs, respectively.

### 5.39 Pseudogene Identification

Pseudogenes were identified using TOGA (v1.1.7) [171], with the human genome assembly hg38 as the reference. Whole‐genome pairwise alignments were first generated using LASTZ (v1.04.22), followed by construction of co‐linear alignment chains using axtChain. RepeatFiller was applied to recover alignments in repetitive regions, and chainCleaner was used to improve alignment specificity. The resulting cleaned chain files were subsequently used as input for TOGA (v1.1.7) to infer pseudogene annotations.

### 5.40 Functional Enrichment Analysis

Gene Ontology (GO) enrichment analyses were performed for candidate gene sets, including genes located in PUR regions, REGs, PSGs, and significantly expanded gene families. Enrichment analyses were conducted using TBtools‐II (https://github.com/CJ‐Chen/TBtools‐II) [172], with the GO annotations of all genes in T2T‐RSC1.0 by InterProScan (v5.71‐102.0) used as the background. Statistical significance was evaluated using a hypergeometric test. GO enrichment analysis of pseudogenes was performed using Metascape (v3.5.20250701; https://metascape.org/gp/index.html#/main/step1) [173], with significance defined as a Benjamini–Hochberg‐adjusted *P*‐value < 0.05.

### 5.41 Construction of Splenic Cell Atlas of Bat

Single‐cell transcriptomic profiles generated using the 10x Genomics platform were processed independently for each individual. The two individuals were treated as independent biological replicates, and samples from different individuals were not pooled during cell preparation or library construction. Low‐quality cells were removed based on quality metrics including the number of detected genes, total UMI counts, and mitochondrial gene content. After quality control, gene expression matrices were normalized using the LogNormalize method implemented in Seurat (v2) [174]. Datasets from different individuals were integrated using the Seurat integration workflow for downstream clustering and visualization. Because the two samples were collected and processed using the same experimental workflow, no additional batch correction was applied beyond the integration procedure. Individual identities were retained throughout the analysis to assess inter‐individual consistency and preserve individual‐specific biological variation. Dimensionality reduction was performed by PCA, followed by visualization using uniform manifold approximation and projection (UMAP). Cell clustering was conducted using the Louvain algorithm implemented in Seurat (v2). Cells from each compartment were then separately and iteratively clustered until the DEGs that distinguished the resultant clusters were deemed not to be biologically relevant. Due to limited molecular information and few cell‐type markers for bat, we based our cluster annotations on DEGs reported in the literature for human, mouse, and Egyptian rousette [64, 65, 66].

### 5.42 Detection of Immune Responses in Splenocytes Stimulated With Lipopolysaccharide (LPS) and Polyinosinic: Polycytidylic Acid [poly(I:C)]

The splenocytes were seeded into 6‐well flat‐bottom culture plates at a density of 1 × 10^5^ cells per well in 2 mL of complete medium. The immune response could be assessed by stimulating isolated splenic lymphocytes ex vivo with LPS (1 µg/mL) and poly(I:C) (1 µg/mL). To determine the differentially expressed genes responding to stimulation, cells were harvested at 6 h post‐infection for RNA‐sequencing and QPCR detection. All experiments were performed with at least three biological replicates. Data are presented as mean ± standard deviation (SD). Statistical significance between treated groups and the unstimulated control was determined using a one‐way ANOVA followed by Tukey's post‐hoc test. A *p*‐value of less than 0.05 was considered statistically significant.

### 5.43 Statistical Analysis

Statistical methods specific to individual analyses were described in the corresponding sections of the Experimental Section. Unless otherwise specified, biological replicates were treated as independent samples, and sample sizes (*n*) are provided in the corresponding Experimental subsections, figure legends, and Supporting Tables. Data are presented as mean ± standard deviation (SD) where applicable. RNA‐seq read counts were normalized using the median‐of‐ratios method in DESeq2, and differential expression was assessed using a negative binomial generalized linear model with Benjamini–Hochberg FDR correction; genes with adjusted *P* < 0.05 and |log_2_FC| > 1 were considered differentially expressed. For genome‐wide selection analyses, LRTs were performed in PAML, followed by Benjamini–Hochberg FDR correction, with *q* < 0.05 considered significant. Viral Shannon diversity indices were compared among geographic regions using Hutcheson's *t*‐test. For immune stimulation assays, statistical differences were assessed using one‐way ANOVA followed by Tukey's post hoc test, with *P* < 0.05 considered significant. Single‐cell RNA‐seq data from two independent biological replicates were quality‐controlled, normalized, and integrated using Seurat as described above. Statistical analyses were performed using the software packages specified in the corresponding Experimental subsections.

## Author Contributions

Conceptualization: G.H. and F.W. Methodology: G.H., H.X., X.D., X.H., and L.L. Formal analysis: G.H., Y.W., S.Z., L.O., J.T., and S.‐R.L. Investigation: G.H., Y.W., S.Z., L.O., J.T., and Z.W. Resources: F.W., G.H., Z.L., and J.Z. Data curation: G.H., Y.W., S.Z., and L.O. Writing and visualization: G.H., Y.W., S.Z., and L.O. Supervision: G.H. and F.W.

## Ethics Statement

All animal experiments and sample collection procedures were reviewed and approved by the Animal Ethics Committee of Jiangxi Agricultural University (approval number: JXAULL‐20260802) and were conducted in accordance with relevant institutional and national guidelines.

## Conflicts of Interest

The authors declare no conflicts of interest.

## Supporting information




**Supporting File 1**: advs78422‐sup‐0001‐SuppMat.docx.

**Supporting File 2**: advs78422‐sup‐0002‐SuppMat.xlsx.

## Data Availability

All raw sequencing reads, multi‐omics datasets, and the T2T‐RSC1.0 genome assembly generated in this study have been deposited in the National Genomics Data Center, China (https://ngdc.cncb.ac.cn/). Raw sequencing reads are available under GSA accession CRA035388. The T2T‐RSC1.0 genome assembly is available in the Genome Warehouse (GWH) under accession number GWHHKIH00000000.1, associated with BioProject PRJCA053466.
