# Supplementary material for: Gapless Complete Genome and Multi‐Omics Illuminate Immune Adaptation and Host‐Virus Interactions in a Coronavirus Reservoir
Source: Adv Sci (Weinh). 2026 Oct 28:e78422. Online ahead of print. doi: 10.1002/advs.78422 (PMC13715679; doi:10.1002/advs.78422)
Supplement: Supplementary file 1 [file ADVS-9999-e78422-s002.docx]

Supplementary Figure

# Gapless complete genome and multi-omics illuminate immune adaptation and host–virus interactions in a coronavirus reservoir

*Yuting Wang^1^, Sheng Zhang^1^, Linxia Ouyang^1^, Xin Du^2,3^, Xin Huang^1^, Zhilin Wang^1^, Huixian Xie^1,2,3^, Jiale Tang^1^, Liyi Lou^1,2,3^, Wenqing Ling^1^, Weihao Wang^1^, Jiang Zhou^4^, Zhen Liu^5^, Yibo Hu^2,3^, Fuwen Wei^1,2,3*^, Guangping Huang^1*^*

^1^ Jiangxi Provincial Key Laboratory of Conservation Biology, College of Forestry, Jiangxi Agricultural University, Nanchang 330045, China

^2^ State Key Laboratory of Animal Biodiversity Conservation and Integrated Pest Management, Institute of Zoology, Chinese Academy of Sciences, Beijing 100101, China

^3^ University of Chinese Academy of Sciences, Beijing 100049, China

^4^ School of Karst Science, Guizhou Normal University, Guiyang 550001, China

^5^ State Key Laboratory of Genetic Evolution & Animal Models, Kunming Institute of Zoology, Chinese Academy of Sciences, Kunming 650201, China

^*^ Correspondence: huanggp@ioz.ac.cn (G.-P.H.), weifw@ioz.ac.cn (F.-W.W.)

Yuting Wang^1^, Sheng Zhang^1^ and Linxia Ouyang^1^ contributed equally to this work.

## Figure S1. Chromosome-level validation of the T2T-RSC1.0 assembly. (A) Hi-C contact heatmaps for all chromosomes, showing chromatin interaction patterns used to assess chromosome-scale assembly accuracy and detect potential misassemblies. (B) Alignment of Bionano optical maps to the 19 chromosomes of T2T-RSC1.0. Blue regions indicate consistent alignments, grey lines denote collinearity, yellow regions mark breaks in collinearity, and green regions represent T2T-RSC1.0–specific sequences lacking enzyme labeling sites. Light blue regions indicate Bionano-specific sequences without enzyme labeling sites.

## Figure S2. Integrated genomic features of the T2T-RSC1.0 genome. Chromosome-scale tracks summarizing major genomic features across the 19 T2T-RSC1.0 chromosomes. Sequencing coverage from HiFi, ONT, and NGS data is shown in mint green, aqua blue, and lavender, respectively (100-kb windows). Repeat density (pale blue), gene density (pink), previously unresolved regions (PURs) relative to the reference genome (rose pink), segmental duplications (pale blue), DNA methylation levels (grayish blue), centromeres (plum), and telomeres (AACCCT motif) are indicated. Topologically associating domains (TADs) inferred from Hi-C data are shown below each chromosome.

## Figure S3. K-mer–based genome characterization and assembly graph of T2T-RSC1.0. (A) K-mer spectra analysis and GenomeScope2 modeling used to estimate genome properties and evaluate assembly quality. The genome size is estimated at ~1.90 Gb, with a heterozygosity of 0.92%, repeat content of 1.02%, sequencing coverage of ~29.4×, an error rate of 0.22%, and ~89.1% non-repetitive sequences. (B) Genome assembly graph of T2T-RSC1.0 visualized from the GFA file. Nodes represent unitigs and edges indicate their connections, outlining candidate chromosome paths.

## Figure S4. Structural variation, completeness, and sequence uniqueness of the T2T-RSC1.0 genome. (A) Genome-wide map of chromosomal structural variations identified by comparison with the reference genome, including inversions, translocations, deletions, and insertions. (B) BUSCO completeness assessment of the T2T-RSC1.0 genome based on the mammalia_odb10 gene set, showing 98.6% complete conserved genes. (C) Distribution of minimum unique k-mers (MUKs) across all chromosomes in 100-kb windows; lower values indicate more repetitive regions, whereas higher values reflect greater sequence uniqueness. (D) Genome-wide distribution of minimum unique k-mer length across chromosomes, with lower values corresponding to higher repeat content and higher values indicating greater sequence uniqueness.

## Figure S5. Assembly and evaluation pipeline for the T2T-RSC1.0 genome. Raw HiFi, ONT, and NGS sequencing data were subjected to quality control and assembled using long-read and hybrid assembly strategies to generate preliminary assemblies. Assembly quality was evaluated, followed by decontamination and Hi-C–based chromosome-scale scaffolding. Gaps were filled, telomeres were resolved, and the assembly was polished and manually inspected to ensure base-level accuracy. The final T2T genome was evaluated using multiple complementary metrics, including completeness, sequence accuracy, coverage, and minimum unique k-mer (MUK) analyses.

## Figure S6. Population genomic analyses of Rhinolophus sinicus. (A) Comparison of insertions and deletions (InDels) between T2T-RSC1.0 and RSC01 across populations, highlighting structural variation. (B) Maximum-likelihood phylogeny (left) and ADMIXTURE analysis (right) based on whole-genome SNPs, with sample lineages indicated by color (SE: bright blue, MW: amber blue, SW: diamond blue). Bootstrap support values are shown at the nodes. The three major genetic groups were each supported by 100% bootstrap support. (C) Principal component analysis (PCA) based on whole-genome and Y-chromosome SNPs. (D) Demographic history inferred by PSMC, showing three glaciation periods: Xixiabangma Glaciation (XG), Penultimate Glaciation (PG), and Last Glacial Maximum (LGM). (E) Genetic differentiation among SE, MW, and SW populations based on pairwise FST. SE and MW show the lowest differentiation (FST = 0.44), while SW is more differentiated from SE (FST = 0.59) and MW (FST = 0.54), indicating pronounced genetic distinctness of the SW group.

## Figure S7. Multi-dimensional features of the Y chromosome in T2T-RSC1.0. (A) Compositional distribution of repetitive sequences on the Y chromosome, with different colors representing distinct repeat types. (B) Triangular self-alignment heatmap of the Y chromosome centromeric region (32.70-33.75 Mb), showing sequence identity levels; inset shows the distribution of identity percentages. (C) Track plot of ATAC-seq and CUT&Tag (H3K27ac, H3K27me3, H3K4me3) signals in the centromeric region, with green, purple, light pink, and teal tracks representing ATAC-seq, H3K27ac, H3K4me3, and H3K27me3 signals, respectively. (D) RNA-seq expression profiles of genes in the Y chromosome ampliconic region across multiple tissues. Light blue squares indicate expressed genes; gray squares with crosses indicate absent expression. Numbers on the left denote gene copy numbers.

## Figure S8. Expression of DEFA5 in HEK293T cells. Bright-field (showing cell morphology) and fluorescence images of HEK293T cells transfected with DEFA5. Green fluorescence indicates successful expression of DEFA5. Scale bar: 50 mm.

## Figure S9. Functional enrichment of differentially expressed genes in mouse and bat. (A) GO enrichment heatmap of DEGs in mouse across different treatment groups (mCtrl_mLPS, mCtrl_mPoly(I:C), mLPS_mPoly(I:C)). Color intensity represents enrichment significance. (B) GO enrichment heatmap of DEGs in bat across treatment groups (bCtrl_bLPS, bCtrl_bPoly(I:C), bLPS_bPoly(I:C)), highlighting immune-related functions such as viral response and type I interferon pathways.
